# Posterolateral or Direct Lateral Surgical Approach for Hemiarthroplasty After a Hip Fracture: A Randomized Clinical Trial Alongside a Natural Experiment

**DOI:** 10.1001/jamanetworkopen.2023.50765

**Published:** 2024-01-11

**Authors:** Maria C. J. M. Tol, Nienke W. Willigenburg, Ariena J. Rasker, Hanna C. Willems, Taco Gosens, Martin J. Heetveld, Martijn G. M. Schotanus, Bart Eggen, Mate Kormos, Stéphanie L. van der Pas, Aad W. van der Vaart, J. Carel Goslings, Rudolf W. Poolman

**Affiliations:** 1 Department of Orthopedic Surgery, Joint Research, OLVG Hospital, Amsterdam, the Netherlands; 2 Department of Internal Medicine and Geriatrics, Amsterdam UMC, Amsterdam, the Netherlands; 3 Department of Orthopedics and Trauma Surgery, ETZ, Tilburg, the Netherlands; 4 Department of Medical and Clinical Psychology, Tilburg University, Tilburg, the Netherlands; 5 Department of Trauma Surgery, Spaarne Gasthuis, Haarlem, the Netherlands; 6 Department of Orthopedic Surgery & Traumatology, Zuyderland Medical Center, Heerlen, Sittard-Geleen, the Netherlands; 7 School of Care and Public Health Research Institute, Faculty of Health, Medicine and Life Science, Maastricht University, the Netherlands; 8 Delft University of Technology, Electrical Engineering, Mathematics and Computer Science, Delft, the Netherlands; 9 Amsterdam UMC location Vrije Universiteit Amsterdam, Epidemiology and Data Science, Amsterdam, the Netherlands; 10 Amsterdam Public Health, Methodology, Amsterdam, the Netherlands; 11 Department of Trauma Surgery, OLVG Hospital, Amsterdam, the Netherlands; 12 Department of Orthopedic Surgery, LUMC, Leiden, the Netherlands

## Abstract

**Importance:**

Hip fractures in older adults are serious injuries that result in disability, higher rates of illness and death, and a substantial strain on health care resources. High-quality evidence to improve hip fracture care regarding the surgical approach of hemiarthroplasty is lacking.

**Objective:**

To compare 6-month outcomes of the posterolateral approach (PLA) and direct lateral approach (DLA) for hemiarthroplasty in patients with acute femoral neck fracture.

**Design, Setting, and Participants:**

This multicenter, randomized clinical trial (RCT) comparing DLA and PLA was performed alongside a natural experiment (NE) at 14 centers in the Netherlands. Patients aged 18 years or older with an acute femoral neck fracture were included, with or without dementia. Secondary surgery of the hip, pathological fractures, or patients with multitrauma were excluded. Recruitment took place between February 2018 and January 2022. Treatment allocation was random or pseudorandom based on geographical location and surgeon preference. Statistical analysis was performed from July 2022 to September 2022.

**Exposure:**

Hemiarthroplasty using PLA or DLA.

**Main Outcome and Measures:**

The primary outcome was health-related quality of life 6 months after surgery, quantified with the EuroQol Group 5-Dimension questionnaire (EQ-5D-5L). Secondary outcomes included dislocations, fear of falling and falls, activities of daily living, pain, and reoperations. To improve generalizability, a novel technique was used for data fusion of the RCT and NE.

**Results:**

A total of 843 patients (542 [64.3%] female; mean [SD] age, 82.2 [7.5] years) participated, with 555 patients in the RCT (283 patients in the DLA group; 272 patients in the PLA group) and 288 patients in the NE (172 patients in the DLA group; 116 patients in the PLA group). In the RCT, mean EQ-5D-5L utility scores at 6 months were 0.50 (95% CI, 0.45-0.55) after DLA and 0.49 (95% CI, 0.44-0.54) after PLA, with 77% completeness. The between-group difference (−0.04 [95% CI, −0.11 to 0.04]) was not statistically significant nor clinically meaningful. Most secondary outcomes were comparable between groups, but PLA was associated with more dislocations than DLA (RCT: 15 of 272 patients [5.5%] in PLA vs 1 of 283 patients [0.4%] in DLA; NE: 6 of 113 patients [5.3%]) in PLA vs 2 of 175 patients [1.1%] in DLA). Data fusion resulted in an effect size of 0.00 (95% CI, −0.04 to 0.05) for the EQ-5D-5L and an odds ratio of 12.31 (95% CI, 2.77 to 54.70) for experiencing a dislocation after PLA.

**Conclusions and Relevance:**

This combined RCT and NE found that among patients treated with a cemented hemiarthroplasty after an acute femoral neck fracture, PLA was not associated with a better quality of life than DLA. Rates of dislocation and reoperation were higher after PLA. Randomized and pseudorandomized data yielded similar outcomes, which suggests a strengthening of these findings.

**Trial Registration:**

ClinicalTrials.gov Identifier: NCT04438226

## Key Points

**Question:**

Is there a difference in patient outcomes between the posterolateral approach (PLA) and direct lateral approach (DLA) for cemented hemiarthroplasty after acute femoral neck fracture?

**Findings:**

In this randomized clinical trial of 555 patients and natural experiment including 288 patients, quality of life 6 months after trauma did not differ between surgical approaches. PLA was associated with significantly more dislocations and reoperations than DLA.

**Meaning:**

This combined randomized clinical trial and natural experiment found no difference in patient-reported quality of life between PLA and DLA for cemented hemiarthroplasty, despite higher rates of dislocation and reoperation after PLA.

## Teaser

This randomized clinical trial compares 6-month outcomes of the posterolateral approach and direct lateral approach for hemiarthroplasty in patients with acute femoral neck fracture.

## Introduction

Hip fractures in older adults are disabling injuries that increase morbidity and mortality,^1^ and cause excessive utilization of health care resources.^2^ High-quality evidence to improve hip fracture care is emerging. The HEALTH trial^3^ showed no clinically meaningful differences between hemiarthroplasty and total hip arthroplasty in the treatment of hip fractures in older adults. The WHITE trial^4^ showed a modestly but significantly better health-related quality of life (HRQOL) and a lower risk of periprosthetic fractures in favor of cemented compared with uncemented hemiarthroplasty. Such evidence is lacking for the choice of surgical approach.

Surgical approaches for hemiarthroplasty vary widely around the globe. The choice of approach is usually determined by the surgeon’s preference or by the agreements made within a hospital. The posterolateral approach (PLA) and the direct lateral approach (DLA) are currently most used.^5,6^ A systematic review on outcomes most relevant for patients suggested that the PLA might be associated with advantages compared with DLA in HRQOL, abductor insufficiency, and walking problems.^7^ A meta-analysis concluded that the PLA had no advantages that counterbalanced its increased risk of dislocation and reoperation compared with DLA and the direct anterior approach.^8^ However, both systematic reviews were based primarily on observational studies and concluded that high-quality clinical trials were needed to confirm or refute their conclusions.

To compare the HRQOL and other relevant patient outcomes between DLA and PLA in adult patients with a displaced femoral neck fracture, we conducted the Surgical Approaches of Cemented Hemiarthroplasty After Hip Fractures Posterolateral vs Direct Lateral Approach (APOLLO) trial. We hypothesized better HRQOL in patients treated with the PLA.

## Methods

This combined randomized clinical trial (RCT) and natural experiment (NE) was approved by the Medical Research Ethics Committees United and the local institutional review boards of all participating centers. All participants provided written informed consent. The steering committee consisted of 3 independent (orthopedic) trauma surgeons who evaluated the interim analysis. This study is reported following the Consolidated Standards of Reporting Trials (CONSORT) reporting guideline.

### Trial Design

The APOLLO trial was a multicenter, randomized superiority trial in the Netherlands with an NE and economic evaluation alongside. Details on the trial objectives, design, procedures, and statistical analysis plan can be found in the published protocol^9^ and Supplement 1.

### Trial Oversight

Fourteen centers participated, of which 9 were only in the NE (eAppendix 1 in Supplement 2). Randomization at the individual patient level occurred when orthopedic surgeons could perform the DLA and the PLA.

The NE was conducted in specific hospitals where surgeons specialized in either the PLA or DLA techniques, and the group of surgeons opted for 1 of these approaches as their standard of care. Given their specialization, these surgeons did not have the flexibility to randomize between the 2 methods. A patient’s proximity to a hospital at the time of the incident (and thus where they were brought to) dictated the surgical approach they would receive, as hospitals were implicitly designated as either DLA or PLA based on the expertise of their surgeons. This geographical-based allocation was outside of research parameters and control. We postulated that this setup mirrored a pseudorandom allocation mechanism, and we rigorously verified this presumption prior to integrating our data.

### Participants

Inclusion criteria were as follows: adult patients (age ≥18 years) with an acute femoral neck fracture (<7 days), cemented hemiarthroplasty as recommended treatment according to the national guidelines, Dutch or English fluency and literacy, and written informed consent. Exclusion criteria were as follows: multitrauma (Injury Severity Score >15), secondary surgery after failed internal fixation, pathological fracture, and high risk of nonadherence (ie, no Dutch residency, such as tourists or patients with a life expectancy <6 months). Cognitive impairment, such as dementia, was not an exclusion criterion.

### Intervention, Randomization, and Blinding

Experienced surgeons or residents under direct supervision of an experienced surgeon performed all operations. Surgical details (ie, whether the piriformis muscle was spared or reattached with the PLA, and how the gluteus medius muscle was closed with the DLA) were left to the surgeon’s discretion.

We used Castor Electronic Data Capture, an online secured data management system with built-in randomization (variable block method, stratified per center), to randomly assign patients in a 1:1 allocation ratio to either PLA or DLA. Surgeons, patients, and outcome assessors were aware of the assignment group because the different surgical approaches were easily distinguishable (ie, based on the scar’s location). Data analysts and the steering committee were blinded. We interpreted the blinded results before breaking the randomization code.

### Outcomes

The primary outcome was the HRQOL as reported by the patient or proxy at 6 months using the EuroQol Group 5-Dimension (EQ-5D-5L). Proxies of incapacitated patients were asked to rate how they thought the patient would rate their HRQOL.^10^ The patients’ EQ-5D-5L health states were converted to utility values (ranging from −0.446 to 1, with higher scores indicating higher health utility) using the Dutch tariff.^11^ Deceased patients had a score of 0.^4,12^

Secondary outcomes are listed in eTable 1 in Supplement 2. We assessed the EuroQol Visual Analog Scale (EQ-VAS; range, 0-100; higher scores indicating a better health status), Katz activities of daily living (ADL) functionality ranging from 0 (independent) to 6 (dependent), and a 5-item mobility score ranging from 0 (no walking aids) to 5 (no functionality of lower extremity). We also considered dichotomous Katz ADL (independent: 0-1 point; dependent: 2 to 6 points) and mobility scores (good: with or without 1 crutch; impaired: more walking aids). Patients also scored their mean and maximum pain during the week using the numeric rating scale ranging from 0 to 10, with higher scores indicating worse pain. We assessed the fear of falling with the Falls Efficacy Scale International (FES-I), ranging from 16 (no concern about falling) to 64 (severe concern about falling). We also recorded the actual fall incidents and additional injuries resulting from falling.

### Assessments

We obtained the outcomes through questionnaires online, by hardcopy, or by phone at 3 and 6 months. In addition, we checked all patients’ medical records up to 6 months postoperatively to record relevant baseline and surgical characteristics, as well as any complications, readmissions, or reoperations during the study period.

### Statistical Analysis

#### Sample Size

To detect a minimally clinically important difference of 0.08 in the primary outcome (EQ-5D-5L utility score)^13^ with an SD of 0.3, we needed 555 patients based on a 2-sided significance level of α = .05 with 80% power and a loss to follow-up of 20% after 6 months.^9^

#### Primary Outcome

We used linear mixed-model analysis to investigate the difference in the primary outcome (EQ-5D-5L utility score) between both surgical approaches. The primary analysis was based on the intention-to-treat principle, with additional as-treated analyses to quantify the effect of protocol deviations. For the crude analyses, the fixed factors were treatment allocation (DLA vs PLA) and the EQ-5D-5L utility score at baseline. We evaluated differences between groups over time by adding time and a time by treatment interaction. Repeated measures within patients and groups of patients within hospitals were clustered using random intercepts. For the adjusted analyses, we added the potential confounders of age, sex, living status, dementia, American Society of Anesthesiologists classification, body mass index, mobility (good vs impaired), and Katz ADL (dependent vs independent) to the model as fixed factors.

#### Secondary Outcomes

The continuous secondary outcomes, fear of falling on the FES-I, functionality on the Katz ADL, and pain on the numeric rating scale were analyzed using similar linear mixed models. Categorical and dichotomous secondary outcomes (ie, mobility, discharge destination, complications, ≥1 fall incidents, additional injuries as a result of falling, or reoperations) were compared using a χ^2^ or Fisher exact test. Statistical analysis was performed from July 2022 to September 2022. We used SPSS version 27.0 (IBM Corp) and a 2-tailed value of α < .05 was considered statistically significant for all analyses.

#### Data Fusion

Besides unconfoundedness, which was assumed, for data fusion it is necessary that there are no significant differences between the patients in the NE and the RCT in any of the treatment groups when correcting for the confounders. We tested this condition on 1 separately imputed combined data set in a manner similar to Lu et al.^14^ We fitted a linear model for the EQ-5D-5L index at 6 months as a function of an experiment indicator and the confounding variables, separately in both groups of surgical approaches. The experiment indicator was 0 for RCT and 1 for NE. If the estimated coefficient belonging to the experiment indicator was close to 0 with *P* > .05, it indicated no statistically significant effect of the experiment indicator on the outcome while keeping all other confounders constant.

We used the augmented inverse probability weighting estimator to determine the average treatment effect between the 2 surgical approaches. We corrected for possible confounders and performed a sensitivity analysis of our chosen methods. Multiple imputation was used on all confounders.^15^ Estimates of the average treatment effect were pooled according to the Rubin rule to form a single estimate and a 95% CI. For more information, see the published protocol^9^ and eMethods in Supplement 2.

## Results

**Figure 1.**
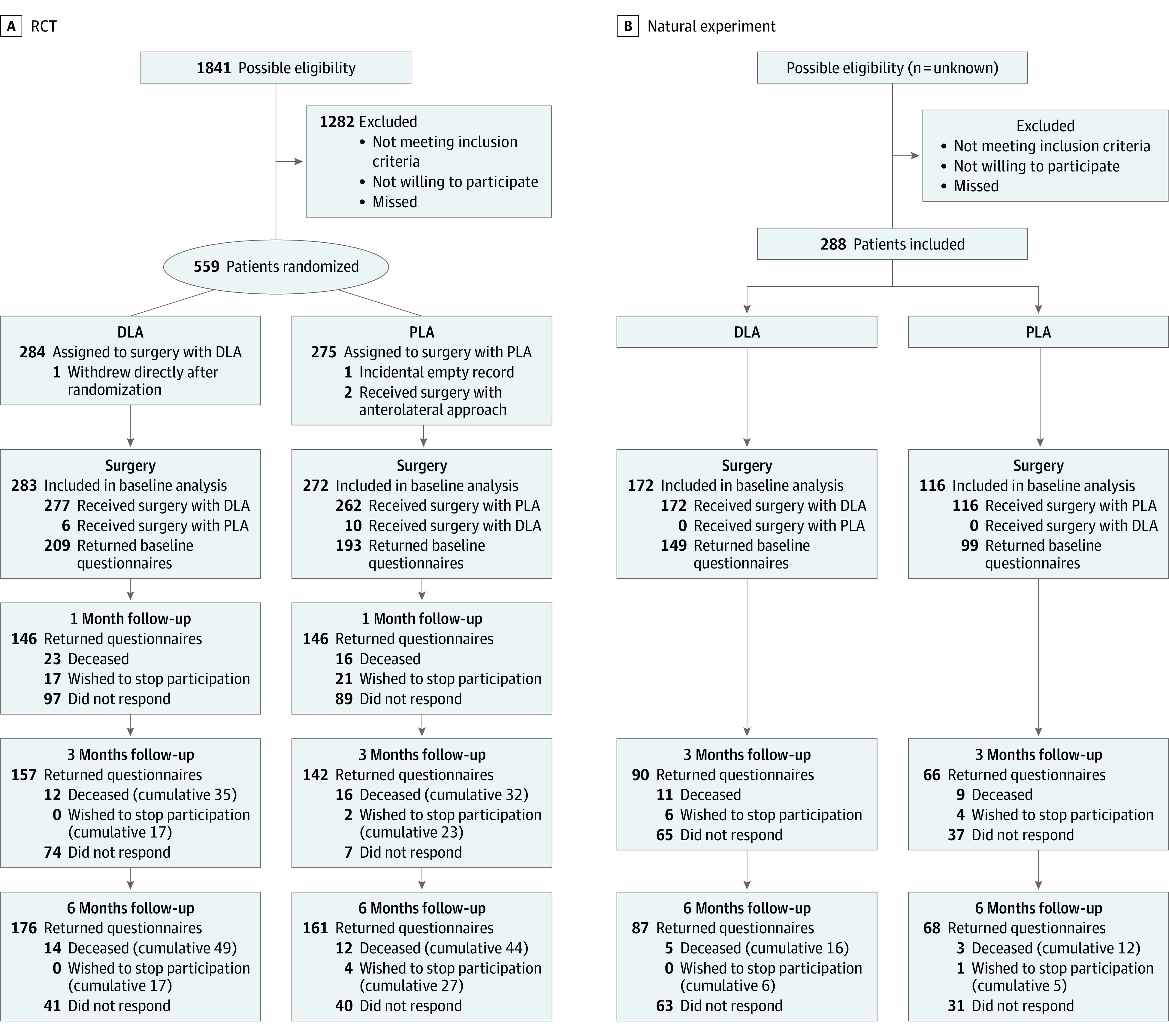
Study Flow Diagrams DLA indicates direct lateral approach; PLA, posterolateral approach; and RCT, randomized clinical trial.

Between February 2018 and January 2022, 843 patients consented to participate (542 [64.3%] female; mean [SD] age, 82.2 [7.5] years). There were 555 patients who participated in the RCT (283 in the DLA group and 272 in the PLA group) and 288 patients in the NE (172 in the DLA group and 116 in the PLA group) (Table). The final 6-month follow-up was completed in July 2022, with complete data for 430 of 555 patients (76.9%) for the primary outcome in the RCT. Figure 1 depicts the patient flow and reasons for exclusion. The percentages of missing data are provided in eFigure 1 in Supplement 2. Nonresponders had a similar age as responders but were less mobile. Patients who dropped out had a similar age as patients who completed the study but were less mobile. Patients with dementia were overrepresented among dropouts in the DLA group (eTable 2 in Supplement 2).

**Table.** Baseline Characteristics of Study Participants

Characteristic	Participants, No. (%) (N = 843)				*P* value of NE
	RCT		NE		
	DLA (n = 283)	PLA (n = 272)	DLA (n = 172)	PLA (n = 116)	
Sex					
Female	172 (60.8)	172 (63.2)	116 (67.4)	82 (70.7)	.42
Male	111 (39.2)	100 (36.8)	56 (32.6)	34 (29.3)	
Age, mean (SD), y	82 (8)	82 (8)	82 (7)	83 (6)	.08
Dementia					
Evident	56 (19.8)	44 (16.2)	20 (11.6)	11 (9.5)	.60
Possibly	18 (6.4)	19 (7.0)	9 (5.2)	9 (7.8)	
ASA classification					
I	7 (2.5)	4 (1.5)	6 (3.5)	6 (5.2)	.78
II	101 (35.7)	80 (29.4)	64 (37.2)	41 (35.3)	
III	152 (53.7)	160 (58.8)	85 (49.4)	56 (48.3)	
IV	10 (3.5)	11 (4.0)	15 (8.7)	7 (6.0)	
BMI, mean (SD)	24.2 (4)	24.6 (4)	24.3 (4)	24.9 (4)	.64
Katz ADL, mean (SD)	1.4 (1.7)	1.3 (1.7)	0.9 (1.4)	0.9 (1.6)	.69
Prescribed medicines, mean (SD), No.	7 (5)	7 (4)	7 (4)	6 (4)	.67
Comorbidities					
Cardiac					
MCI	29 (10.2)	30 (11.0)	22 (12.8)	8 (6.9)	.11
Heart failure	20 (7.1)	20 (7.4)	25 (14.5)	9 (7.8)	.08
Arrhythmia	49 (17.3)	56 (20.6)	58 (33.7)	21 (18.1)	.01
Neurological					
Hemiparalysis	14 (4.9)	8 (2.9)	8 (4.7)	5 (4.3)	>.99
CVA	26 (9.2)	25 (9.2)	18 (10.5)	6 (5.2)	.11
Parkinson	20 (7.1)	15 (5.5)	3 (1.7)	3 (2.6)	.69
Epilepsy	3 (1.1)	2 (0.7)	1 (0.6)	1 (0.9)	>.99
Pulmonal					
COPD	39 (13.8)	46 (16.9)	20 (11.6)	9 (7.8)	.29
Asthma	10 (3.5)	10 (3.7)	6 (3.5)	4 (3.4)	.99
Prefracture mobility					
Without aids	94 (33.2)	99 (36.4)	70 (40.7)	43 (37.1)	.35
1 Crutch	26 (9.2)	27 (9.9)	18 (10.5)	18 (15.5)	
Walker	106 (37.5)	92 (33.8)	53 (30.8)	40 (34.5)	
Outside with help	29 (10.2)	20 (7.4)	18 (10.5)	8 (6.9)	
No mobility	2 (0.7)	5 (1.8)	3 (1.7)	0	
Living status					
Independent	153 (54.1)	149 (54.8)	115 (66.9)	80 (69.0)	.40
Independent with help	61 (21.6)	69 (25.4)	36 (20.9)	15 (12.9)	
Residential care	29 (10.2)	29 (10.7)	9 (5.2)	5 (4.3)	
Nursing home	32 (11.3)	20 (7.4)	7 (4.1)	9 (7.8)	
Rehabilitation unit	3 (1.1)	3 (1.1)	1 (0.6)	2 (1.7)	
Other	0	0	3 (1.7)	2 (1.7)	

Abbreviations: ADL, activities of daily living; ASA, American Society of Anesthesiologists; BMI, body mass index (calculated as weight in kilograms divided by height in meters squared); COPD, chronic obstructive pulmonary disease; CVA, cerebral vascular accident; DLA, direct lateral approach; MCI, myocardial infarct; NE, natural experiment; PLA, posterolateral approach; RCT, randomized clinical trial.

Baseline characteristics were comparable between treatment groups (DLA vs PLA) and between study designs (RCT vs NE), but patients with arrhythmia were overrepresented in the NE-DLA group (Table). Most participants (432 [77.8%] in RCT; 246 [85.4%] in NE) lived independently (with or without ADL help) before the hip fracture. Signs of dementia were present in 137 patients (24.7%) in the RCT and 49 patients (17.0%) in the NE.

### Adherence to the Assigned Intervention

Sixteen protocol deviations occurred. Six patients assigned to DLA (2.1%) had surgery with PLA, and 10 patients assigned to PLA (3.7%) had surgery with DLA (Figure 1).

### RCT Primary End Point

**Figure 2.**
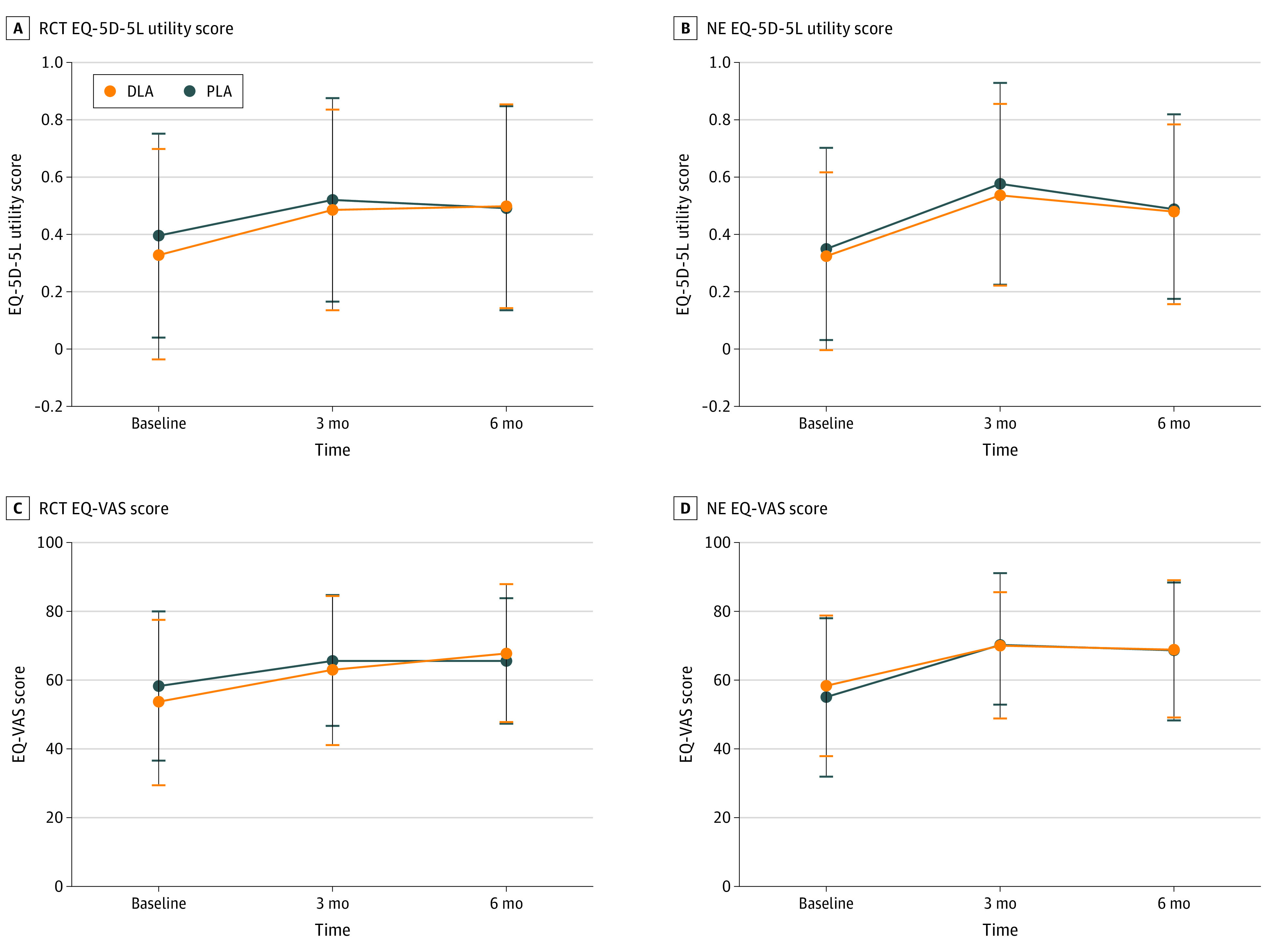
EuroQol Group 5-Dimension (EQ-5D-5L) and EuroQol Visual Analog Scale (EQ-VAS) Outcomes NE indicates natural experiment; RCT, randomized clinical trial.

Mean EQ-5D-5L utility score at the 6-month follow-up was 0.50 (95% CI, 0.45 to 0.55) in the DLA group and 0.49 (95% CI, 0.44 to 0.54) in the PLA group (Figure 2). Intention-to-treat analyses showed no statistically significant between-group difference at 6 months (crude estimate: −0.05 [95% CI, −0.14 to 0.04]; adjusted estimate: −0.04 [95% CI, −0.11 to 0.04]; the negative sign indicates in favor of PLA) (eTable 3 in Supplement 2). As-treated analyses yielded similar between-group difference results (crude estimate: −0.06 [95% CI, −0.15 to 0.03]; adjusted estimate: −0.04 [95% CI, −0.12 to 0.03]).

### RCT Secondary End Points

**Figure 3.**
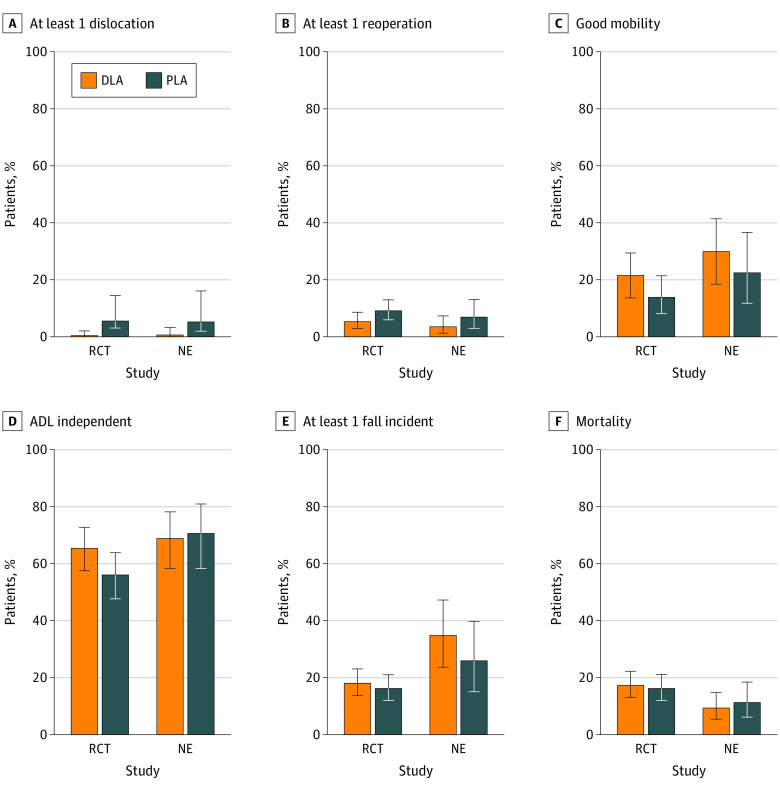
Secondary Outcomes After 6 Months ADL indicates activities of daily living; DLA, direct lateral approach; NE, natural experiment; PLA, posterolateral approach; and RCT, randomized clinical trial.

At the 6-month follow-up, the mean EQ-VAS was 67.85 (95% CI, 65.12 to 71.01) points after DLA and 65.58 (95% CI, 62.78 to 68.38) points after PLA (Figure 2). The quality-adjusted life-years did not differ between the PLA and DLA groups. Figure 3 and eFigure 2 and eTable 3 in Supplement 2 summarize the other secondary outcomes. There were no statistically significant differences in ADL Katz score between the groups, nor in the dichotomized Katz ADL scores. Mean and maximum pain scores were similar after DLA and PLA. At 6 months, 29 patients (21.5%) in the DLA group had good mobility, compared with 16 patients (13.8%) in the PLA group (*P* = .11). The frequency of falls did not differ between the surgical approaches: 51 patients in the DLA group (18.0%) reported at least 1 fall incident vs 44 patients in the PLA group (16.2%) (*P* = .77). There were no differences in the rate of emergency department admissions or additional injuries due to the falls. The fear of falling measured with the FES-I questionnaire was not significantly different between the groups (Figure 3). We observed similar outcomes in both treatment groups regarding the length of stay, surgery time, postoperative complications, and discharge destinations (eTable 4 in Supplement 2).

The rate of prosthesis dislocation was significantly different between groups, both statistically and clinically (eTable 5 in Supplement 2). Dislocation occurred in 15 of 272 patients in the PLA group (5.5%), compared with 1 of 283 patients in the DLA group (0.4%), resulting in an odds ratio of 16.46 (95% CI, 2.16 to 125.48; *P* < .001). Six patients (2.2%) in the PLA group experienced recurrent dislocations, and 8 patients (2.9%) underwent revision surgery. The overall number of reoperations was 35 in the PLA group and 18 in the DLA group (eTable 6 in Supplement 2).

During the 6-month follow-up, death occurred in 93 of 555 patients (16.8%). Mortality was similar in both the DLA and PLA groups. Between-group differences at 3 and 6 months follow-up are detailed in eTable 3 in Supplement 2.

### NE Primary and Secondary End Points

The EQ-5D-5L utility score at 6 months did not differ between the treatment groups (DLA: 0.53 [95% CI, 0.47 to 0.60]; PLA: 0.57 [95% CI, 0.50 to 0.64]; group effect size: 0.06 [95% CI, −0.07 to 0.18]) (Figure 2; eTable 3 in Supplement 2). We found a higher risk of dislocation in the PLA group (6 of 113 patients [5.3%]) compared with DLA (2 of 175 patients [1.1%]) (eTable 4 in Supplement 2). Figure 3 presents all secondary outcomes of the RCT and NE.

### Data Fusion Outcomes

**Figure 4.**
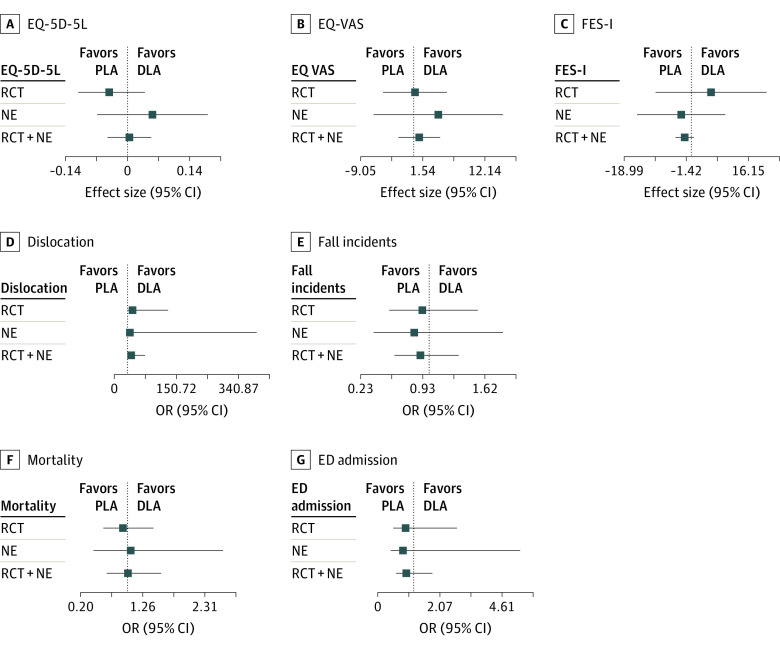
Fused Results of Primary and Secondary Outcomes DLA indicates direct lateral approach; ED, emergency department; EQ-5D-5L, EuroQol Group 5-Dimension questionnaire; EQ-VAS, EuroQol Visual Analog Scale; FES-I, Falls Efficacy Scale International; NE, natural experiment; OR, odds ratio, PLA, posterolateral approach; and RCT, randomized clinical trial. Dotted line indicates 0 (A, B, and C) or 1.00 (D, E, F, and G).

For the primary outcome, there was no significant difference between the groups in the different experiments when all other confounders were included in the model, so no evidence was found against the assumptions for data fusion (eMethods and eAppendix 2 in Supplement 2). Fusion tests were rejected for the Katz ADL score, mobility, pain mean, and pain max variables. Data fusion resulted in an effect size of 0.00 (95% CI, −0.04 to –0.05) for the EQ-5D-5L and an odds ratio of 12.31 (95% CI, 2.77 to 54.70) for experiencing a dislocation after PLA. All estimates, including 95% CIs, are presented in Figure 4.

## Discussion

This RCT conducted alongside an NE found that surgical approach for a cemented hemiarthroplasty after a femoral neck fracture was not associated with the HRQOL in adult patients with an acute hip fracture 6 months after surgery. The observed mean difference in EQ-5D-5L utility score between DLA and PLA was neither statistically significant nor clinically relevant. In addition, the secondary outcomes in function, pain, mobility, and tendency to fall did not differ between the groups. However, patients in the PLA group had a higher risk of prosthesis dislocation and reoperation due to dislocation, which was statistically significant and also clinically relevant.

Our study has several strengths. First, to our knowledge, this is the largest RCT worldwide addressing this topic. Second, the NE that we designed alongside the RCT showed strong potential as a feasible alternative to randomization at the individual patient level. Treatment allocation in the NE was purely based on the geographical location (and thereby the hospital) and the timing of the fracture (and subsequently the surgeon who would perform the hemiarthroplasty). Our study provides important insights in the similarities between RCT and NE study populations and outcomes, which is relevant given the known difficulties with surgical RCTs.^16^ The addition of the NE raised the number of participants and increased the generalizability of our trial results. As we were not restricted to surgeons with expertise in both approaches, more hospitals could participate. Another strength of our study is the inclusion of patients with cognitive impairments, such as dementia. Older adults with dementia are well represented in the population of patients with hip fractures; however, they are often excluded from RCTs.^17^ Including them further increased the generalizability of the results.

### Limitations

There were some limitations to our study. First, missing data were prevalent in the RCT and NE data sets. This was mainly caused by nonresponse to the follow-up questionnaires, which was associated with dementia, living status, and mobility. However, it is essential to include these patients because they are a substantial part of the hip fracture population. Sensitivity analyses with multiple imputation analyses for confounders found no differences between the approaches. There are no missing data nor loss to follow-up for the outcomes of dislocation, reoperation, readmissions, and mortality. For these variables, we reviewed all medical reports 6 months postoperatively and used registry data for possible adverse events in other hospitals. Second, a screening log was not maintained, so we do not formally know why not all eligible patients were randomized. We know that some patients declined participation and not all patients received information about the study due to logistical and time constraints. Our overview of potentially eligible patients based on the Dutch arthroplasty registry^6^ shows that their characteristics were similar to the randomized group. Third, we did not gather information on whether the piriformis muscle was spared or reattached. Currently, there is no high-level evidence for the effect of piriformis-sparing approaches of hemiarthroplasty. Although we acknowledge the potential variability in surgeon expertise and specialty, our study’s outcomes remain consistent across the board. Future studies might benefit from a deeper dive into the influence of surgeon subspecialties on hemiarthroplasty outcomes.

To our knowledge, no RCT to date evaluating the surgical approach for hemiarthroplasty reported the EQ-5D-5L. A large Norwegian observational study showed a significant difference of 0.03 in favor of PLA.^18^ We observed no significant difference in favor of the PLA, but both differences are under the threshold of the minimally clinically important difference of 0.08.^13^ Two other prospective observational studies did not report any differences in the EQ-5D-5L related to the surgical approach after adjusting for confounders.^19,20^

Prosthesis dislocation was more common in the PLA group, with 5.5% vs 0.4% in the DLA group, which was similar in the NE. Thereby, the risk of reoperation due to dislocation was statistically significantly higher in the PLA group (2.9% vs 0%). These findings support the existing evidence of observational studies.^19,21,22,23^ While most clinical trials do not include patients with dementia, we did include this population, which may contribute to a relatively high dislocation rate compared with the existing literature. The higher risk of dislocation rate was seen in both study designs. The potential bias of performance bias in the RCT was not seen in this trial. This increased risk of dislocation did not result in a substantially lower quality of life in the PLA group, which raises the question whether the EQ-5D-5L is sufficiently sensitive to quantify the effectiveness of orthopedic interventions. On the other hand, the more specific patient-reported secondary outcomes, including mobility, fear of falling and falling, and Katz ADL, were also not significantly different between surgical approaches.

## Conclusions

In this RCT alongside an NE, PLA was not associated with a better quality of life compared with DLA among adult patients treated with a cemented hemiarthroplasty after an acute femoral neck fracture. Most secondary outcomes were similar between groups, but PLA was associated with more dislocation and reoperation compared with DLA. Pseudorandomization in our NE resulted in similar outcomes and could be a valid and more feasible alternative to the traditional RCT.

The implications of our results to improve patient care are not straightforward. The increased risk for dislocation and reoperation after PLA without clear benefits of that approach could justify a recommendation for DLA. Alongside this trial, we conducted an economic evaluation, and we thoroughly quantified physical performance and balance in a subgroup of patients. These additional outcomes will help to weigh a broader spectrum of costs and benefits associated with DLA and PLA to better inform evidence-based decisions on the surgical approach for older patients with hemiarthroplasty after a hip fracture.

## Notes

Supplement 1.
Trial Protocol

Supplement 2. **eAppendix 1.** Investigators and Participating Hospitals **eMethods.** Data Fusion **eAppendix 2.** Data Fusion Test for the Primary Outcome EQ-5D-5L Utility Score **eFigure 1.** Missing Values for Relevant Patient Characteristics and Outcomes **eTable 1.** Overview of Outcomes and Measurement Moments **eTable 2.** Baseline Characteristics of Lost to Follow-Up Patients for Primary Outcome at Endpoint **eTable 3.** Detailed Between-Group Effects at 3 and 6 Months for Primary and Secondary Outcomes **eFigure 2.** Secondary Outcomes at 6-Months **eTable 4.** Peri- and Post-Operative Outcomes of the RCT **eTable 5.** Dislocation Rates **eTable 6.** Reoperations by Indication **eTable 7.** Subgroup Analyses
eReferences.

Supplement 3.
APOLLO Research Group Members

Supplement 4.
Data Sharing Statement
