# Supplementary material for: Posterolateral or Direct Lateral Surgical Approach for Hemiarthroplasty After a Hip Fracture: A Randomized Clinical Trial Alongside a Natural Experiment
Source: JAMA Netw Open. 2024 Jan 11;7(1):e2350765. doi: 10.1001/jamanetworkopen.2023.50765 (PMC10784859; doi:10.1001/jamanetworkopen.2023.50765)
Supplement: Supplement 1. — Trial Protocol [file jamanetwopen-e2350765-s001.pdf]

"Surgical Approach of Hemiarthroplasty after Femoral Neck Fracture: Posterolateral or Direct Lateral"

APOLLO trial

RESEARCH PROTOCOL

(Version 03)

PROTOCOL TITLE

'Surgical Approach of Hemiarthroplasty after Femoral Neck Fracture: Posterolateral of Direct Lateral'

**Protocol ID**	APOLLO trial
**Short title**	Surgical Approach of Hemiarthroplasty after Femoral Neck Fracture: Posterolateral of Direct Lateral
**Version**	04
**Date**	
**Coordinating investigator/project leader**	M.C.J.M. Tol
**Principal investigator(s) (in Dutch: hoofdonderzoeker/ uitvoerder)**	Dr. R.W. Poolman, *OLVG*<br>Dr. M.J. Heetveld, *Spaarne Gasthuis*<br>Dr. H. Willems, *AMC*<br>Dr. T. Gosens, *Elisabeth-Tweesteden ziekenhuis*<br>Dr. A. Vochteloo, *Ziekenhuisgroep Twente*
**Sponsor (in Dutch: verrichter/opdrachtgever)**	Raad van bestuur OLVG
**Subsidising party**	ZonMw<br>Nederlandse Orthopaedische Vereniging
**Independent expert**	Dr. M.P.J. van den Bekerom<br>m.vandenbekerom@olvg.nl

PROTOCOL SIGNATURE SHEET

Name	Signature	Date
Head of Department:	Dr. R.W. Poolman	
[Coordinating Investigator/Project leader/Principal Investigator]:	Dr. R.W. Poolman	

TABLE OF CONTENTS

1. INTRODUCTION AND RATIONALE ... 9
2. OBJECTIVES ... 10
3. STUDY DESIGN ... 11
4. STUDY POPULATION ... 11
4.1 Population (base) ... 11
4.2 Inclusion criteria ... 11
4.3 Exclusion criteria ... 11
4.4 Sample size calculation ... 11
5. TREATMENT OF SUBJECTS ... 12
5.1 Investigational treatment ... 12
6. METHODS ... 13
6.1 Study parameters/endpoints ... 13
6.1.1 Main study parameter/endpoint ... 13
6.1.2 Secondary study parameters/endpoints ... 13
6.2 Randomisation, blinding and treatment allocation ... 13
6.3 Natural Experiment ... 14
6.4 Study procedures ... 15
6.5 Withdrawal of individual subjects ... 17
6.6 Replacement of individual subjects after withdrawal ... 17
6.7 Follow-up of subjects withdrawn from treatment ... 17
6.8 Premature termination of the study ... 18
7. SAFETY REPORTING ... 18
7.1 Temporary halt for reasons of subject safety ... 18
7.2 AEs and SAEs ... 18
7.2.1 Adverse events (AEs) ... 18
7.2.2 Serious adverse events (SAEs) ... 18
7.3 Follow-up of serious adverse events ... 19
7.4 Data Safety Monitoring Board (DSMB) / Safety Committee ... 19
8. STATISTICAL ANALYSIS ... 20
8.1 Primary study parameter(s) ... 20
8.2 Secondary study parameter(s) ... 20
8.3 Cost-effectiveness analyses ... 20
9. ETHICAL CONSIDERATIONS ... 21
9.1 Regulation statement ... 21
9.2 Recruitment and consent ... 21
9.3 Objection by minors or incapacitated subjects (if applicable) ... 21
9.4 Benefits and risks assessment, group relatedness ... 22
9.5 Compensation for injury ... 22
9.6 Incentives ... 22
10. ADMINISTRATIVE ASPECTS, MONITORING AND PUBLICATION ... 23
10.1 Handling and storage of data and documents ... 23

10.2 Monitoring and Quality Assurance 23
10.3 Amendments 23
10.4 Annual progress report 23
10.5 Temporary halt and (prematurely) end of study report 23
10.6 Public disclosure and publication policy 24
11. STRUCTURED RISK ANALYSIS 25
12. REFERENCES 25

LIST OF ABBREVIATIONS AND RELEVANT DEFINITIONS

**ABR**	**ABR form, General Assessment and Registration form, is the application form that is required for submission to the accredited Ethics Committee (In Dutch, ABR = Algemene Beoordeling en Registratie)**
**ADL**	**Activities of Daily Living**
**AE**	**Adverse Event**
**APOLLO**	**surgical Approach of hemiarthroplasty after femoral neck fracture: POsteroLateraL Or direct Lateral**
**CCMO**	**Central Committee on Research Involving Human Subjects; in Dutch: Centrale Commissie Mensgebonden Onderzoek**
**CV**	**Curriculum Vitae**
**DHFA**	**Dutch Hip Fracture Audit**
**DSMB**	**Data Safety Monitoring Board**
**DLA**	**Direct Lateral Approach**
**EQ-5D-5L**	**Health related quality of life**
**FES-I**	**The Falls Efficacy Scale-International**
**GEE**	**Generalized Estimating Equation**
**IC**	**Informed Consent**
**ICERs**	**Incremental Cost Effectiveness Ratios**
**ISS**	**Injury Severity Score**
**KATZ**	**Katz Activities of Daily Living**
**MCID**	**Minimal Clinical Important Difference**
**MEC-U**	**Medical research Ethics Committees United**
**METC**	**Medical research ethics committee (MREC); in Dutch: medisch ethische toetsing commissie (METC)**
**NE**	**Natural Experiment**
**NRS**	**Numeric Rating Scale**
**OLVG**	**Onze Lieve Vrouwe Gasthuis**
**PLA**	**Posterolateral Approach**
**PIF**	**Patient Information Form**
**QALY**	**Quality Adjusted Life Year**
**RCT**	**Randomised Controlled Trial**
**SAE**	**Serious Adverse Event**
**SPPB**	**Short Physical Performance Battery balance test**
**SPPS**	**Statistical Package for the Social Sciences**

**Sponsor**	**The sponsor is the party that commissions the organisation or performance of the research, for example a pharmaceutical company, academic hospital, scientific organisation or investigator. A party that provides funding for a study but does not commission it is not regarded as the sponsor, but referred to as a subsidising party.**
**Wbp**	**Personal Data Protection Act (in Dutch: Wet Bescherming Persoonsgevens)**
**WMO**	**Medical Research Involving Human Subjects Act (in Dutch: Wet Medisch-wetenschappelijk Onderzoek met Mensen**

SUMMARY

Rationale: In the Netherlands the two main surgical approaches for hemiarthroplasty are the posterolateral and the direct lateral approach**.** Currently there is no conclusive evidence which of these two approaches results in better patient outcomes.

Objective: Assessing the patient outcome comparing the posterolateral with the direct lateral approach in patients being treated with cemented hemiarthroplasty after femoral neck fractures.

Study design: A randomised controlled multi-center superiority trial and natural experiment with an economic evaluation alongside.

Study population: All patients older than 18 years with a femoral neck fracture whereby treatment with cemented hemiarthroplasty is recommended according the national guidelines.

Intervention: Treatment with cemented hemiarthroplasty using the posterolateral approach.

Standard intervention to be compared to: Treatment with cemented hemiarthroplasty using the direct lateral approach.

Main study parameters/endpoints: The primary outcome is the patient-rated quality of life (EQ-5D-5L) at 6 months after surgery.
Secondary outcomes are: ADL functionality (KATZ), Balance test (SPPB), Tendency to Fall (FES-I), Pain (NRS), Re-interventions, Mobility, Discharge destination, Complications, and cost-effectiveness.

Nature and extent of the burden and risks associated with participation, benefit and group relatedness: The different approaches in the two treatment arms of the randomised controlled trial are widely used techniques in the Netherlands and many of the outcome measures are part of the standard clinical follow-up after hip fracture. Therefore, there is no extra risk or burden for participating patients, except for the time to complete some additional follow-up measurements. The primary outcome measurement and secondary outcomes, will be assessed through questionnaires online, by hardcopy or by phone at baseline, 3 and 6 months postoperatively. The assessment of the Short Physical Performance Battery (SPPB) balance test, will be performed by one of the study researchers or nurse practitioner to protect continuity and feasibility.

1. INTRODUCTION AND RATIONALE

Annually approximately 21.000 patients are admitted to a hospital in the Netherlands with a hip fracture(1). It is the most devastating fracture in the elderly and is associated with excessive utilization of health care resources. The tendency to fall is prominent among elderly and increases with age and frailty level. After a first hip fracture, patients are at increased risk for recurrent falls and prone for additional injuries. This results in impairment of postoperative rehabilitation and increased morbidity and total health-care costs(2). Furthermore, the increased morbidity strongly affects the patients' health related quality of life (HRQoL)(3).
In the treatment of hip fractures, hemiarthroplasty is a commonly used procedure. Different surgical techniques are described in the literature. However, in the Netherlands the two main surgical approaches for hemiarthroplasty are the posterolateral lateral and the direct lateral approach(4).

When inserting a hemiarthroplasty through the posterolateral approach (PLA), the surgeon performs a posterior capsulotomy. During the PLA the hip abductors are protected and preserved preventing limping. However, due to inadequate posterior capsule support there is an increased risk of dislocations(5–10). The direct lateral approach (DLA) involves an incision of the gluteus medius and vastus lateralis muscles. This frequently leads to abductor insufficiency resulting in limping after surgery(11,12). However, in the DLA the posterior capsule is preserved preventing dislocation.

Currently there is no conclusive evidence of which of these two approaches results in the better patient outcomes. The PLA is assumed to be beneficial regarding HRQoL and some studies report that patients operated with PLA are more satisfied and experience less pain after a hemiarthroplasty (13–15). The presumably faster rehabilitation and better balance due to the scatheless gluteus musculature in patients treated using the PLA may be counterbalanced by the increased risk of dislocation. On the contrary, the loss of abductor muscle strength after hemiarthroplasty through the DLA can hypothetically lead to a loss of balance which intensifies the risk of falling and can result in less mobile patients. The increased immobility in the already vulnerable elderly patients will lead to higher risk of falling, asks more of caregivers and patients will rely more on home care facilities.

Hip fracture patients can become more vulnerable and less dependent after subsequent falls. There is still much to gain in terms of reducing these risk factors. Therefore, to detect the differences in surgical approach and improve the patient outcomes is paramount. Well conducted clinical trials comparing the two different – most frequently used in the Netherlands - surgical approaches are absent. Therefore, it remains unknown whether hip fracture patients should be treated using the PLA or the DLA for a cemented hemiarthroplasty.

HEALTH CARE EFFICIENCY PROBLEM

Currently the Dutch national guidelines recommend either the anterior or the anterolateral approach, despite the lack of high quality evidence. The anterior approach gained popularity – without high level supporting evidence - in elective total hip arthroplasty for osteoarthritis. However, the anterior approach is only used in 3 percent of all hemiarthroplasties in the acute setting, whereas the PLA and DLA are used in respectively 50% and 35% in the treatment of hip fractures with hemiarthroplasty(4). This illustrate the excessively high variance in practice between surgeons and need for high quality evidence.

Moreover, as stated above there is an absence of evidence which surgical approach is most beneficial for the patient outcome. Despite the slightly higher risk of dislocation there is a trend to better patient reported outcomes after hemiarthroplasty using the PLA in terms of satisfaction, pain and HRQoL compared with the DLA group(13–15). Thereby, the loss of gluteal muscle strength results in less stability, more limping and can hypothetically lead to a higher tendency to fall which is related with additionally injuries and a prolonged rehabilitation(2). All the more rehabilitation remains the main cost determinant after inserting a hemiarthroplasty(16).

The existing knowledge gap which surgical approach is preferable for the patient outcome and the present lack of continuity, gives us a window of opportunity to improve the quality of life and health care for patients with hip fractures treated with a hemiarthroplasty.

2. OBJECTIVES

Primary Objective:

- Does hemiarthroplasty using the posterolateral approach result in a superior patient-reported quality of life compared to the direct lateral approach in the treatment of femoral neck fractures?

Secondary Objectives:

- Does a hemiarthroplasty using the posterolateral approach lead to a reduction in health care related costs compared to the direct lateral surgical approach?

- Does a hemiarthroplasty using the posterolateral approach lead to better patient outcomes regarding: ADL functionality, balance, tendency to fall, pain, mobility, the number of re-interventions or complications and the discharge destination.

3. STUDY DESIGN

A randomised controlled multicenter superiority trial and natural experiment with an economic evaluation alongside. This study will be conducted in the Netherlands and aims for completion within 36 months.

4. STUDY POPULATION

4.1 Population (base)

The population base includes all consecutive patients of both sexes and all ethnicities with fractures of the proximal femur admitted to the emergency room or surgery/orthopaedic department of the participating hospitals in the Netherlands during the inclusion period (estimated December 2017 - September 2020).

4.2 Inclusion criteria

In order to be eligible to participate in this study, a subject must meet all of the following criteria:

- ≥ 18 years at time of trauma
- Acute hip fracture
- Hemiarthroplasty as recommended treatment according the national guidelines
- Dutch or English fluency and literacy
- Informed consent or by proxy in patients with mental impairment

4.3 Exclusion criteria

A potential subject who meets any of the following criteria will be excluded from participation in this study:

- Multi-trauma-patient (ISS > 15)
- Secondary surgery after failed internal fixation
- Patients with a known metastatic disease and a confirmed pathological fracture of the hip
- Fracture > 7 days at time of surgery
- High risk of non-compliance/adherence to study procedures (e.g. no Dutch residency during follow-up period, or other factors that impair follow-up data collection)

4.4 Sample size calculation

We based our sample size calculation on a superiority design. As stated before the EQ-5D will serve as primary outcome measure. We have used the results from earlier studies on this subject for our sample size calculation(17–19). For the sample size calculation, we hypothesized that the EQ-5D will be higher in the PLA group. Using a two-sided significance level (a) of 0.05 and a power (ß) of 80% with a standard deviation (SD) of 0.3 and a minimal clinical important difference (MCID) of 0.08 (derived from the study mentioned before) a total of 222 subjects are needed in each treatment arm. Taking into account a 25% loss to follow-up after six months, a total number of 610 participants are needed in this superiority design.

5. TREATMENT OF SUBJECTS

5.1 Investigational treatment

Patients allocated to the PLA group will receive a cemented hemiarthroplasty using the PLA.

Technique classic PLA

Patient in side position. Exposure is made through a 10-15 cm curved incision that begins about 7 cm cranial and posterior of the greater trochanter and continue across the greater trochanter down towards the femur shaft. In line with the skin incision, the fascia lata is incised to exposure the distal vastus lateralis. The gluteus maximus muscle is separated by blunt dissection. Stretch the short external rotators and detach the piriformis and obturator internus muscle. Expose the hip joint by a longitudinally incision of the posterior capsule. Dislocate the hip with internal rotation after capsulotomy.(20)

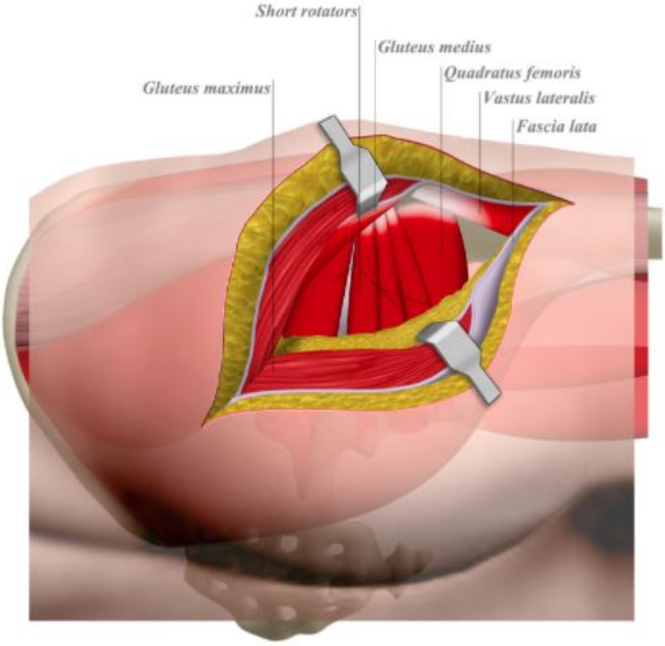

The type and brand of the prosthesis will be left to the surgeons' discretion. Hospital protocols and guidelines will be followed regarding physical therapy and rehabilitation program and will be recorded.

Patients allocated to the DLA group will be treated with a cemented hemiarthroplasty using the DLA.

Technique classic DLA

Patient in supine position. Exposure is made through a longitudinal incision beginning about 5 cm proximal and continuing over the tip of the greater trochanter and extends down the line of the femur shaft for 8 cm. Incise fascia lata in line with the skin incision. Retract the fascia lata anteriorly and the gluteus maximus posteriorly. Blunt dissection of any fibers of gluteus medius that attach to fascia lata. Insertion of the gluteus medius through a crescent shaped course 3-5 cm above the greater

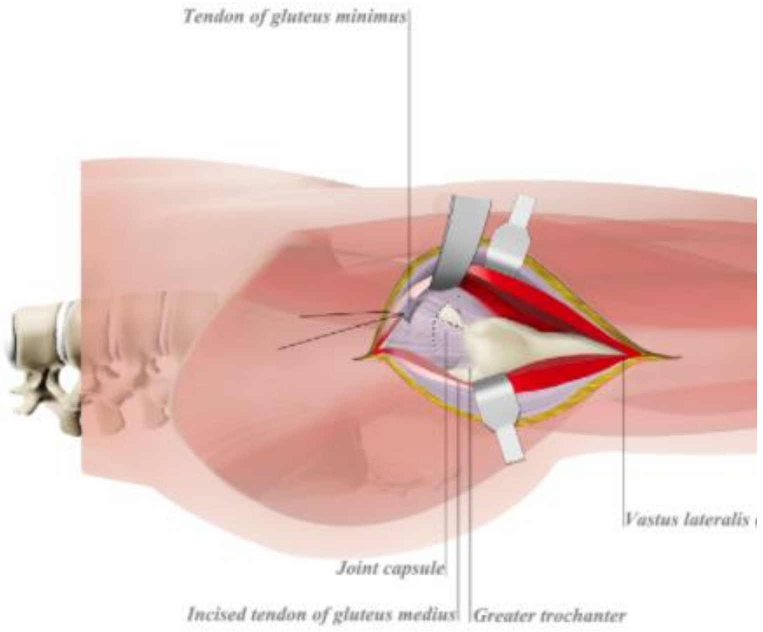

trochanter and extend the incision inferior through the fibers of vastus lateralis. Develop an anterior flap by sharp dissection of anterior gluteus medius fibers and the anterior part of the vastus laterals of the bone. Exposure of the hip joint is reached by releasing the gluteus minimus from the antererior greater trochanter to expose and dissect the anterior joint capsule.(21)

The type and brand of the prosthesis will be left to the surgeons' discretion and implant specifications will be recorded. Physical therapy and rehabilitation will be administered following the standard protocols and guidelines from the center of inclusion.

6. METHODS

6.1 Study parameters/endpoints

6.1.1 Main study parameter/endpoint

The primary outcome is the patient (or proxy)-rated quality of life (EQ-5D-5L) at 6 months after surgery.

6.1.2 Secondary study parameters/endpoints

Secondary outcomes are: ADL functionality (KATZ), Balance test (SPPB), Tendency to Fall (FES-I), Numbers of falls, additional injuries as a result of falling, Pain (NRS), Re-interventions, Mobility (pre-fracture mobility score), Discharge destination, (surgical) complications and cost-effectiveness.

6.2 Randomisation, blinding and treatment allocation

Eligible patients admitted to the hospitals where both surgical techniques are practiced, are invited for participating in our RCT prior to the surgery. When patients agree to participate, written informed consent will be obtained and patients will be randomised between the PLA and the DLA. Randomisation will be stratified per center and per orthopaedic/trauma surgeon. Randomisation will be done in CASTOR EDC, a secured study and data management system with built-in randomisation (variable block method).

Surgeons, patients or outcome assessors cannot be blinded since the different surgical approaches are easily distinguishable (i.e. based on the location of the scar).

6.3 Natural Experiment

Alongside the RCT we will conduct a natural experiment in hospitals with surgeons who are only comfortable (or competent) in performing one of the two surgical approaches. Allocation to treatment using the PLA or DLA is determined by the hospital where the patient is admitted to (i.e. by the topographical location where the trauma takes place). These natural factors are outside the control of the investigators, resembling random assignment. Patients in the natural experiment are invited for participating after surgery, and informed consent will be obtained for follow-up and data usage.
We will recruit participating centers based on surgical expertise balancing number of centers based on surgical approaches used.
Although we are aware this is not a formal randomization on participant level, the NE design has several advantages(22):

- Most importantly, we will prevent surgical expertise bias.
- The NE design will facilitate better generalizability of our trial results since more centers are able to participate. Generalizability will help implementing our trials results.
- We will be able to reduce selection bias, by including patients and surgeons who may not have agreed to randomisation.

Interventions to prevent other forms of bias:

- Selection bias: We will be able to compare the RCT and NE results with anonymous data from the DHFA registry that includes all patients who were treated for hip fractures in the participating centers.
- Detection bias: Patients participating in our study will be blinded to the study hypothesis.
- Attrition bias: Follow-up will be performed by a research coordinator assuring completeness of data.
- Reporting bias: We will publish our study protocol in an open access peer reviewed journal and at www.clinicaltrials.gov
- Performance bias: The Dutch Hip Fracture Guideline introduced in 2015 will prevent performance bias since treatment protocols are standardised apart from the surgical approach used. Hip fracture guideline adherence is good (23).

6.4 Study procedures

	Screening	*Informed consent procedure*	*Baseline assessment*	*Surgery*	*4 weeks follow up*	*3 months follow up*	*6 months follow up*
Clinician/ researcher	Identify eligible patients with hip fractures	Explain PIF, answer questions, sign ICF	DHFA*	Record implant details<br>Record complications		Standard clinical follow up, including DHFA	Standard clinical follow up
Researcher	Identify eligible patients with hip fractures	Answer additional questions if needed	- DHFA<br>- EQ-5D	Screen surgical report for relevant information if necessary	Health care utilization question-naire	- EQ-5D<br>- FES-1 scale<br>- NRS<br>- SPPB<br>- Health care utilization questionnaire	- EQ-5D<br>- FES-1 scale<br>- NRS<br>- DHFA<br>- Health care utilization questionnaire
Patient/ proxy		Read PIF, ask questions, sign IC	Complete questionnaire		Complete question-naire	- Complete questionnaires<br>- SPPB at outpatient clinic	Complete questionnaires

*DHFA is a registry used in standard care includes: living status, pre-mobility score, KATZ, ASA, complications, discharge destination, re-operations

When hospitals are participating with this trial, treating physicians will be asked to screen if patients admitted to the hospital with a hip fracture are eligible for study entry. If inclusion criteria are met, they can contact the research coordinator by phone (☎ (+31) 650 568 721) or visit the trial website, for further assistance with obtaining informed consent, randomisation and inclusion of the patient.

To obtain informed consent, the patient information letter will be handed out to eligible patients or to their health care proxy. Hard copies will be available in all participating hospitals or could be find as a download on the trials' website.
Hip fractures are injuries where treatment is required in less than 48 hours for optimal outcomes. Therefore, the time in which patients can consider if they are willing to participate to the study, is limited to the time untill surgery. Informed consent of patients in the Natural Experiment can be obtained after surgery. The research coordinator will contact these patients.

For patients with severe cognitive impairment due to dementia, informed consent will be obtained by their health care proxy. It is to the doctors' opinion to determine if a patient

has a cognitive impairment. When the diagnosis is not clear, a clinical geriatric doctor will be consulted

Randomisation

After obtaining informed consent at the emergency department or patient ward, patients will be randomly assigned in a 1:1 allocation ratio to one of the following study arms:

- Treatment with a cemented hemiarthroplasty using the DLA
- Treatment with a cemented hemiarthroplasty using the PLA

Baseline assessment

At baseline we will assess the EQ-5D-5L, the ADL functionality (KATZ), pre-mobility score and the living status using the Dutch Hip Fracture Audit (DHFA) registration. The assessment of the above outcomes is based on the health status prior to the trauma.

Surgery

All operations will be performed by experienced surgeons or residents under the direct supervision of an experienced surgeon. Antibiotic and tromboembolic prophylaxis and wound dressing are done according to the judgment of participating surgeon and local guidelines. The surgical report will be available to the research team in order to extract any relevant information on the procedure. Hospital protocols and guidelines will be followed regarding physical therapy and rehabilitation program, and will be recorded. The DHFA will be completed after surgery and after check-up in the outpatient clinic.

Follow-up data collection

The baseline and follow-up assessment will be conducted by the coordinating research team of the OLVG. There are three follow-up moments at 4 weeks, 3 and 6 months postoperative. The primary outcome measurement EQ-5D-5L and the secondary outcomes FES-I scale will be assessed through questionnaires online, by hardcopy or by phone at baseline, 3 and 6 months postoperatively. The health care utilization questionnaire will be assessed at 4 weeks, 3 and 6 months follow-up. The questionnaires will be handed out by the coordinating researcher. Other secondary outcome measurements (e.g. KATZ, mobility score, complications, re-operations) are detailed in the DHFA at baseline and 3 months post-operatively and will be assessed through questionnaires online, by hardcopy or by phone at 6 months after surgery and will be handed out by the coordinating researcher.

The assessment of the Short Physical Performance Battery (SPPB) balance test, which is not included in the DHFA, will be performed by one of the study researchers or nurse practitioner to protect continuity and feasibility and will only be assessed in patients included in the RCT. The SPPB will not be assessed in all patients, but in a subgroup of the patient population because the sample size is smaller. The SPPB is a group of measures that combines the results of the gait speed, chair stand and balance tests.(24)

The follow-up questionnaires will contain:

- Health-related quality of life as measured by the EQ-5D questionnaire. The EQ-5D-5L is a descriptive system of health-related quality of life states consisting of five dimensions (mobility, self-care, usual activities, pain/discomfort and anxiety/depression)(25).
- The Katz Index of Independence in Activities of Daily Living, the Katz ADL, is an instrument to assess functional status as a measurement of the client's ability to perform activities of daily living independently consisting six functions (bathing, dressing, toileting, transferring, continence and feeding)(26).
- The Falls Efficacy Scale-International (FES-I) is a short, easy to administer tool that measures the level of concern about falling during social and physical activities inside and outside the home whether or not the person actually does the activity(27).
- Additionally injuries as a result of falling and other (indication to) re-interventions
- Mobility is assessed by the Pre-fracture Mobility scale, which is specific for hip fracture patients. The scale represent patients with no need for any walking aid and no restriction in walking distance to patients who are bedbound(28).
- Pain as measured by a eleven-point Numeric Rating Scale(29).
- Health care resources utilization (including amongst others, number of visits to the general practitioner and use of home care organizations)

6.5 Withdrawal of individual subjects

Subjects can leave the study at any time for any reason if they wish to do so without any consequences. The investigator can decide to withdraw a subject from the study for medical or other reasons. Data collected prior to individual subject withdrawal will be used for data analysis, unless the patient requests otherwise.

6.6 Replacement of individual subjects after withdrawal

We do not intend to replace individual subjects after withdrawal, as we anticipated a 25% of lost to follow-up. If the drop-out rate appears to exceed this estimation, we will reconsider.

6.7 Follow-up of subjects withdrawn from treatment

If patients – for whatever reason – do not undergo the allocated treatment, this will be recorded as protocol deviation. Withdrawing from the randomized treatment is not a reason to terminate study participation, because follow up data of these patients are needed for intention-to-treat analyses. Therefore, patients will be invited for the study follow up measurements, and details of the treatment they received instead of the randomized procedure will be recorded. Subjects will be invited for all follow up measurements as described in this protocol, except those who explicitly withdraw informed consent.

6.8 Premature termination of the study

Any considerations to terminate this trial prematurely will be discussed with the research team, the sponsor, the subsidizing parties and the MEC-U.

6.9 Missing subjects

Subject can be missed during screening for study entry and subjects can refuse to participate in the study. Whether there are differences in baseline characteristics between the missing subjects and included subjects, data from the DHFA registration is collected. Of all consecutive patients treated with a hemiarthroplasty after femoral neck fracture admitted in one of the participating hospitals during the inclusion period, the following variables will be collected: age, gender, ASA, BMI, cognitive impairment and date of death.

7. SAFETY REPORTING

7.1 Temporary halt for reasons of subject safety

In accordance to section 10, subsection 4, of the WMO, the sponsor will suspend the study if there is sufficient ground that continuation of the study will jeopardise subject health or safety. The sponsor will notify the accredited METC without undue delay of a temporary halt including the reason for such an action. The study will be suspended pending a further positive decision by the accredited METC. The investigator will take care that all subjects are kept informed.

7.2 AEs and SAEs

7.2.1 Adverse events (AEs)

Adverse events are defined as any undesirable experience occurring to a subject during the study, whether or not considered related to the surgical approach. All adverse events that are potentially related to the intervention and reported spontaneously by the subject or observed by the investigator or his staff will be recorded.

7.2.2 Serious adverse events (SAEs)

A serious adverse event is any untoward medical occurrence or effect that

- results in death;
- is life threatening (at the time of the event);

- requires hospitalisation or prolongation of existing inpatients' hospitalisation;
- results in persistent or significant disability or incapacity; or
- any other important medical event that did not result in any of the outcomes listed above due to medical or surgical intervention but could have been based upon appropriate judgement by the investigator.

An elective hospital admission will not be considered as a serious adverse event.

The investigator will report all SAEs related to the treatment to the sponsor without undue delay after obtaining knowledge of the events, except for the following SAEs; SAEs that are unrelated to the study intervention.

The sponsor will report the SAEs through the web portal *ToetsingOnline* to the accredited METC that approved the protocol, within 7 days of first knowledge for SAEs that result in death or are life threatening followed by a period of maximum of 8 days to complete the initial preliminary report. All other SAEs will be reported within a period of maximum 15 days after the sponsor has first knowledge of the serious adverse events.

7.3 Follow-up of serious adverse events

All SAEs related to the intervention will be followed until they have abated, or until a stable situation has been reached. Depending on the event, follow up may require additional tests or medical procedures as indicated, and/or referral to the general physician or a medical specialist.
SAEs need to be reported till end of study within the Netherlands, as defined in the protocol

7.4 Data Safety Monitoring Board (DSMB) / Safety Committee

Not applicable, as both study interventions are common procedures in the Netherlands.

8. STATISTICAL ANALYSIS

Descriptive analysis will be performed to compare baseline characteristics between the patients treated using the PLA and DLA. We will use generalized estimating equations (GEE) for longitudinal analysis on an intention-to-treat and additional per-protocol basis to investigate the effect of surgical approach. All analyses will be done using the Statistical Package for the Social Sciences (SPSS, Chicago, Illinois, USA).

8.1 Primary study parameter(s)

In the primary GEE model, the primary outcome variable studied (health related quality of life on EQ-5D) will be analyzed as a dependent variable, using treatment allocation (DLA vs. PLA) as between subjects' variable and time as within subjects' variable. In a similar way, continuous secondary outcome variables will be analyzed using similar GEE models. All models will assess the interaction term of group and time, to evaluate whether the change over time differed between PLA and DLA.

8.2 Secondary study parameter(s)

Dichotomous secondary outcome measures will be analyzed using chi square statistics and logistic regression models. Subgroup analysis will be performed for patients with dementia. For all analyses, a two-tailed value of $p < 0.05$ is considered to be significant.

8.3 Cost-effectiveness analyses

An economic evaluation will be performed form the societal perspective and in accordance with the intention-to-treat principle. All costs and consequences relevant to the intervention will be taken into account. Intervention costs will be estimated using a micro-costing approach. Retrospective cost questionnaires will be administered at 4 weeks, and at 3 and 6 months follow-up to collect data on healthcare utilization (for example visits to general practitioner, physiotherapy, exercise therapy, rehabilitation, social work, medication), the use of informal care, absenteeism, presenteeism, and unpaid productivity losses. Resource use will be valued in accordance with the Dutch manual of costing of the Netherlands Health Institute (Zorginstituut Nederland). Missing data will be imputed using multiple imputation by changed equations(30). Incremental Cost Effectiveness Ratios (ICERs) will be calculated by dividing the difference in costs by that in effects. We will perform a cost-utility analysis with QALYs as outcome. In order to account for the possible clustering of data, analyses will be performed using linear multilevel analyses(31). Accounting for the possible clustering of data (e.g. at the hospital level) is very important, as most economic evaluations fail to do so, whereas

ignoring the possible clustering of data might lead to inaccurate levels of uncertainty and inaccurate point estimates(31). Bootstrapping techniques will be used to estimate the uncertainty surrounding the cost-effectiveness estimates. Uncertainty will be shown in cost-effectiveness planes and cost-effectiveness acceptability curves, and sensitivity analyses will be performed to test the robustness of the study results(32–34).

9. ETHICAL CONSIDERATIONS

9.1 Regulation statement

The study will be conducted according to the principles of the Declaration of Helsinki, as amended in Seoul and Fortaleza (64th WMA General Assembly, October 2013)(35) and in accordance with the Medical Research Involving Human Subjects Act (WMO) and other guidelines, regulations and Acts.

9.2 Recruitment and consent

Every patient with a hip fracture which should be treated with a hemiarthroplasty will be invited for study entry. Information will be given through the patient information letter and patients are able to ask questions to the treating physician or investigator. Their consent will be asked by the treating physician. In case of incapacitated subjects (i.e. elderly with cognitive impairment such as dementia), informed consent will be received by proxy through the legally representative or a family member. A copy of the signed informed consent form will be given to the patient/proxy as a separate document together with the patient information letter. Until the moment of surgery, patients will have the time to reconsider their decision. Normally, patients with hip fractures are treated in 24 hours after admission to the emergency room.

9.3 Objection by minors or incapacitated subjects (if applicable)

This study is a therapeutic research and will include incapacitated subjects, elderly with a cognitive impairment such as dementia. Whether the patient is participating with this trial or not, all patients who are eligible for this study have to undergo surgery as treatment for the hip fracture. In case of incapacitated subjects informed consent will be received by proxy through the legally representative or a family member. In the unlikely event that incapacitated subject explicitly object to participate, they can leave the study without any further consequences.

9.4 Benefits and risks assessment, group relatedness

The different approaches in the two treatment arms of the randomised controlled trial are widely used techniques in the Netherlands. At this moment the geographical location where the trauma takes place, will determine in which hospital the patient is operated and which surgical approach they will use. Therefore, there are no extra risks of burden for participating patients. For the patient there will be no large direct benefit of participating to this study. However, the direct lateral approach may leads to a loss of balance due to abductor insufficiency and therefore might increase the risk of falling. This can result in reduced mobility and more vulnerability, asking more of caregivers and/or patients will relying more on home care facilities. The potential value of this research is to maintain the independency of vulnerable elderly patients after hip fractures.

Currently there is a lack of studies investigating the quality and efficiency of care for the elderly and the organization of care, due to the fact that frail elderly patients are frequently excluded in clinical trials. Therefore, the generalizability of most studies is limited and the validity of many guidelines for elderly are inadequate. We think it is paramount to include the frail elderly patients, as they are the majority of the patients who sustain a hip fracture. In our study protocol we followed the guideline for medical research in the elderly(36), that gives guidance to research in this frail patient population.

This guideline concludes that results will be more relevant to medical care for elderly patients, if vulnerability and comorbidity in itself are no longer reasons to exclude patients. Therefore, we think including frail elderly patients is a major asset in our clinical trial.

9.5 Compensation for injury

The sponsor/investigator has a liability insurance which is in accordance with article 7 of the WMO. The surgery techniques in both treatment arms are widely used in the Netherlands, therefore we will apply for exemption for the insurance for subjects participating in medical research at the consent committee.

9.6 Incentives

Travel expenses incurred by the participating patients who are visiting the outpatient clinic an extra time are compensated and these costs are budgeted in the financial plan of the study.

10. ADMINISTRATIVE ASPECTS, MONITORING AND PUBLICATION

10.1 Handling and storage of data and documents

All data will be handled according to a data management plan. The multicenter study will generate new quantitative data, which we will be kept for 15 years. The source data will be archived and managed at our academy, the OLVG. All included patients receive a trial code, which anonyms their personal data. The link between the trial code and the patient personal data is saved on a separate secured file with access only by the coordinating investigator.

The handling of personal data will be complied with the Dutch Personal Data Protection Act

10.2 Monitoring and Quality Assurance

A monitoring plan is currently being developed at OLVG (initiating center), and will be applied to this project when ready. Study monitoring will be done using a risk-based strategy.

10.3 Amendments

Amendments are changes made to the research after a favourable opinion by the accredited METC has been given. All amendments will be notified to the METC that gave a favourable opinion. All substantial amendments will be notified to the METC and to the competent authority. Non-substantial amendments will not be notified to the accredited METC and the competent authority, but will be recorded and filed by the sponsor.

10.4 Annual progress report

The sponsor/investigator will submit a summary of the progress of the trial to the accredited METC once a year. Information will be provided on the date of inclusion of the first subject, numbers of subjects included and numbers of subjects that have completed the trial, serious adverse events/ serious adverse reactions, other problems, and amendments.

10.5 Temporary halt and (prematurely) end of study report

The investigator/sponsor will notify the accredited METC of the end of the study within a period of 8 weeks. The end of the study is defined as the last patient’s last visit.

The sponsor will notify the METC immediately of a temporary halt of the study, including the reason of such an action.

In case the study is ended prematurely, the sponsor will notify the accredited METC within 15 days, including the reasons for the premature termination.
Within one year after the end of the study, the investigator/sponsor will submit a final study report with the results of the study, including any publications/abstracts of the study, to the accredited METC.

10.6 Public disclosure and publication policy

The APOLLO trial is a collaboration between the Dutch Orthopaedic, Trauma and Geriatric association. We will sign a consortium agreement with all parties concerning the public disclosure and publication of the research date. Our study protocol and results of the study will be published in an open access peer reviewed journal, according to the principles of the publication policy of the CCMO. Thereby, we will register our trial at www.clinicaltrials.gov.

11. STRUCTURED RISK ANALYSIS

Non applicable

12. REFERENCES

1. Richtlijn Proximale Femurfractuur. 2016.
2. Burgers PTPW, Zielinski SM, Mailuhu AKE, Heetveld MJ, Verhofstad MHJ, Roukema GR, et al. Cumulative incidence and treatment of non-simultaneous bilateral femoral neck fractures in a cohort of one thousand two hundred and fifty patients. Int Orthop. 2014;38(11):2335–42.
3. Gjertsen J-E, Baste V, Fevang JM, Furnes O, Engesæter LB. Quality of life following hip fractures: results from the Norwegian hip fracture register. BMC Musculoskelet Disord. BMC Musculoskeletal Disorders; 2016;17(1):265.
4. LROI. Chirurgische benadering kophalsprothese.
5. Biber R, Brem M, Singler K, Moellers M, Sieber C, Bail HJ. Dorsal versus transgluteal approach for hip hemiarthroplasty: An analysis of early complications in seven hundred and four consecutive cases. Int Orthop. 2012;36(11):2219–23.
6. Rogmark C, Fenstad AM, Leonardsson O, Engesæter LB, Kärrholm J, Furnes O, et al. Posterior approach and uncemented stems increases the risk of reoperation after hemiarthroplasties in elderly hip fracture patients. Acta Orthop. 2014;85(1):18–25.
7. Mukka S, Mahmood S, Kadum B, Sköldenberg O, Sayed-Noor A. Direct lateral vs posterolateral approach to hemiarthroplasty for femoral neck fractures. Orthop Traumatol Surg Res. Elsevier Masson SAS; 2016;102(8):1049–54.
8. Ozan F, Öncel ES, Koyuncu Ş, Gürbüz K, Doğar F, Vatansever F. Effects of Hardinge versus Moore approach on postoperative outcomes in elderly patients with hip fracture. 2016;9(2):4425–31.
9. Paton RW, Hirst P. Hemiarthroplasty of the hip and dislocation. Injury. 1989;20(3):167–9.
10. Unwin AJ, Thomas M. Dislocation after hemiarthroplasty of the hip: A comparison of the dislocation rate after posterior and lateral approaches to the hip. Ann R Coll Surg Engl. 1994;76(5):327–9.
11. Berstock JR, Blom AW, Beswick AD. A systematic review and meta-analysis of complications following the posterior and lateral surgical approaches to total hip arthroplasty. Ann R Coll Surg Engl. 2015;97(1):11–6.
12. Sayed-noor AS, Hanas A, Sköldenberg OG, Mukka SS. Abductor Muscle Function and Trochanteric Tenderness After Hemiarthroplasty for Femoral Neck Fracture. 2016;194–200.
13. Kristensen TB, Vinje T, Havelin LI, Engesæter LB. Posterior approach compared to

direct lateral approach resulted in better patient-reported outcome after hemiarthroplasty for femoral neck fracture Posterior approach compared to direct lateral approach resulted in better patient-reported outcome after hemiarthro- plasty for femoral neck fracture 20 , 908 patients from the Norwegian Hip Fracture Register. 2016;3674(January 2017).

14. Amlie E, Havelin LI, Furnes O, Baste V, Nordsletten L, Hovik O, et al. Worse patient-reported outcome after lateral approach than after anterior and posterolateral approach in primary hip arthroplasty A cross-sectional questionnaire study of 1 , 476 patients 1 – 3 years after surgery. 2014;85(5):463–9.
15. Leonardsson O, Rolfson O, Rogmark C. The surgical approach for hemiarthroplasty does not influence patient-reported outcome. 2016;542–7.
16. Burgers PTPW, Hoogendoorn M, Woensel EAC Van. Total medical costs of treating femoral neck fracture patients with hemi- or total hip arthroplasty : a cost analysis of a multicenter prospective study. Osteoporos Int [Internet]. Osteoporosis International; 2016;1999–2008. Available from: http://dx.doi.org/10.1007/s00198-016-3484-z
17. Parsons N, Achten J, Griffin XL, Costa ML, Reed MR. The World Hip Trauma Evaluation Study 3. Bone Jt Res. 2016;5:18–25.
18. Walters S, Brazier J. Comparison of the minimally important difference for two health state utility measures: EQ-5D and SF-6D. Qual Life Res. 2005;14(6):523–32.
19. Hakkaart–Van Roijen L, Tan S, Bouwmans C. No TitleHandleiding voor kostenonderzoek. Methoden en standaard kostprijzen voor economische evaluaties in de gezondheidszorg. Geactualiseerde versie 2010. 2010.
20. Moore A. Metal hip joint: a new selflocking vitallium prosthesis. South Med J. 1952;45(11):1015–9.
21. Hardinge K. The direct lateral approach to the hip. J Bone Joint Surg Br. 1982;64(1):1017–9.
22. Mahabier K, Van Lieshout E, Bolhuis H, Bos P, Bronkhorst M, MM B, et al. - HUMeral shaft fractures: measuring recovery after operative versus non-operative. ANZ J Surg. 2014;84(4):218–24.
23. Zielinski SM, Meeuwis MA, Heetveld MJ, Verhofstad MHJ, Roukema GR, Patka P, et al. Adherence to a femoral neck fracture treatment guideline. Int Orthop. 2013;37(7):1327–34.
24. Guralnik J, Simonsick E, Ferrucci L, Glynn R, Berkman L, Blazer D, et al. A short physical performance battery assessing lower extremity function: association with self-reported disability and prediction of mortality and nursing home admission. J Gerontol. 1994;49(2):85–94.
25. Rabin R, de Charro F. EQ-5D: a measure of health status from the EuroQol Group.

Ann Med. 2001;33(5):337–43.
26. Katz S. Assessing self-maintenance: activities of daily living, mobility, and instrumental activities of daily living. J Am Geriatr Soc. 1983;31(12):721–7.
27. Yardley L, Beyer N, Hauer K, Kempen G, Piot-Ziegler C, Todd C. Development and initial validation of the Falls Efficacy Scale-International (FES-I). Age Ageing. 2005;34(6):614–9.
28. Bowers T, Parker M. Assessment of outcome after hip fracture: development of a universal assessment system for hip fractures. SICOT J. 2016;2(27).
29. McCaffery M, Beebe A. Pain: Clinical Manual for Nursing Practice. 1993.
30. White I, Royston P, Wood A. Multiple imputation using chained equations: Issues and guidance for practice. Stat Med. 2011;30:377–99.
31. Gomes. Methods for covariate adjustments in cost-effectiveness analysis that use cluster randomised trials. Heal Econ. 2012;21(9):1108–18.
32. Black W. The CE plane: a graphic representation of cost-effectiveness. Med Decis Mak. 1990;10:212–4.
33. Fenwick E. Cost-effectiveness acceptability curves. Heal Econ. 2004;13:405–15.
34. Drummond M, Sculpher M, Torrance G, O'Brien B, Stoddart G. Methods for the Economic Evaluation of Health Care Programmes 3rd Edition. Oxford Univ Press. 2005;
35. 64th WMA General Assembly. Declaration of Helsinki. Fortaleza, Brazil. 2013.
36. van der Marck M, Smeulders E, Olde Rikkert M. Leidraad voor medisch-wetenschappelijk onderzoek bij ouderen. 2017.

Appendix 1

Contact information

Coordinating investigator: M.C.J.M. Tol
☎ (+31) 650 568 721
✉ mcjm.tol@gmail.com

Onze Lieve Vrouwe Gasthuis
Orthopedie
Postbus 95500
1090 HM Amsterdam

Principal investigators: Dr. R.W. Poolman
☎ (020) 599 1065
✉ rwp@jointresearch.org

Onze Lieve Vrouwe Gasthuis
Orthopedie
Postbus 95500
1090 HM Amsterdam

Dr. M.J. Heetveld
☎ (023) 224 0000
✉heetveld@spaarnegasthuis.nl

Spaarne Gasthuis
Chirurgie
Boerhaavelaan 22
20135 RC Haarlem

Dr. H.C. Willems

Academisch Medisch Centrum
Inwendige Geneeskunde Geriatrie
Postbus 22660
1100 DD Amsterdam

Local investigators: Dr. T. Gosens

Elisabeth-Tweesteden Ziekenhuis
Orthopedie
Postbus 90151
5000 LC Tilburg

Dr. A. Vochteloo

Ziekenhuisgroep Twente
Orthopedie
Postbus 7600
7600 SZ Almelo
