# Supplementary material for: Posterolateral or Direct Lateral Surgical Approach for Hemiarthroplasty After a Hip Fracture: A Randomized Clinical Trial Alongside a Natural Experiment
Source: JAMA Netw Open. 2024 Jan 11;7(1):e2350765. doi: 10.1001/jamanetworkopen.2023.50765 (PMC10784859; doi:10.1001/jamanetworkopen.2023.50765)
Supplement: Supplement 2. — eAppendix 1. Investigators and Participating Hospitals eMethods. Data Fusion eAppendix 2. Data Fusion Test for the Primary Outcome EQ-5D-5L Utility Score eFigure 1. Missing Values for Relevant Patient Characteristics and Outcomes eTable 1. Overview of Outcomes and Measurement Moments eTable 2. Baseline Characteristics of Lost to Follow-Up Patients for Primary Outcome at Endpoint eTable 3. Detailed Between-Group Effects at 3 and 6 Months for Primary and Secondary Outcomes eFigure 2. Secondary Outcomes at 6-Months eTable 4. Peri- and Post-Operative Outcomes of the RCT eTable 5. Dislocation Rates eTable 6. Reoperations by Indication eTable 7. Subgroup Analyses eReferences. [file jamanetwopen-e2350765-s002.pdf]

Supplemental Online Content

Tol MCJM, Willigenburg NW, Rasker AJ, et al; the APOLLO Research Group. Posterolateral or direct lateral surgical approach for hemiarthroplasty after a hip fracture: a randomized clinical trial alongside a natural experiment. *JAMA Netw Open.* 2024;7(1):e2350765. doi:10.1001/jamanetworkopen.2023.50765

eAppendix 1. Investigators and Participating Hospitals

eMethods. Data Fusion

eAppendix 2. Data Fusion Test for the Primary Outcome EQ-5D-5L Utility Score

eFigure 1. Missing Values for Relevant Patient Characteristics and Outcomes

eTable 1. Overview of Outcomes and Measurement Moments

eTable 2. Baseline Characteristics of Lost to Follow Up Patients for Primary Outcome at Endpoint

eTable 3. Detailed Between-Group Effects at 3 and 6 Months for Primary and Secondary Outcomes

eFigure 2. Secondary Outcomes at 6-months

eTable 4. Peri- and Post-Operative Outcomes of the RCT

eTable 5. Dislocation Rates

eTable 6. Reoperations by Indication

eTable 7. Subgroup Analyses

eReferences

This supplemental material has been provided by the authors to give readers additional information about their work.

© 2024 Tol MCJM et al. *JAMA Network Open.*

Posterolateral or Direct Lateral Surgical Approach

for Hemiarthroplasty after a Hip Fracture

© 2024 Tol MCJM et al. *JAMA Network Open.*

eAppendix 1. Investigators and Participating Hospitals

Investigators of the APOLLO research group (all located in the Netherlands):

- Frank van Roon, M.D. Department of Orthopedics and Trauma Surgery, ETZ, Tilburg
- Martijn van Dijk, M.D., Ph.D. Department of Orthopedic Surgery, Antonius Ziekenhuis, Utrecht
- Jort Keizer, M.D. Department of Trauma Surgery, Antonius Ziekenhuis, Utrecht
- Anne J.H. Vochteloo, M.D., Ph.D. Department of Orthopedic Surgery, OCON, Hengelo
- Pieter Joosse, M.D., Ph.D. Department of Trauma Surgery, Noord-West Ziekenhuis, Alkmaar
- Bert Boonen, M.D., Ph.D., Department of Orthopedic Surgery & Traumatology, Zuyderland Medical Center, Heerlen, Sittard-Geleen
- Jetse Jelsma, M.D., Ph.D., Department of Orthopedic Surgery & Traumatology, Zuyderland Medical Center, Heerlen, Sittard-Geleen
- Dieuwertje Theeuwen, M.D. Department of Orthopedic Surgery & Traumatology, Zuyderland Medical Center, Heerlen, Sittard-Geleen
- Joris J.W. Ploegmakers, M.D., Ph.D. Department of Orthopedic Surgery, UMCG, Groningen
- Tim Schepers, M.D. Ph.D. Department of Trauma Surgery, Amsterdam UMC, Amsterdam
- Evelien van der Meij, M.D. Department of Orthopedic Surgery, IJsselland, Capelle aan den IJssel
- Svenhjalmar H. van Helden, M.D., Ph.D. Department of Orthopedic & Trauma Surgery, Isala, Zwolle
- Rutger Zuurmond, M.D., Ph.D. Department of Orthopedic & Trauma Surgery, Isala, Zwolle
- Bart A. van Dijkman, M.D. Department of Trauma Surgery, Flevoziekenhuis, Almere
- Thomas D. Berendes, M.D., Ph.D. Department of Orthopedic Surgery, Meander MC, Amersfoort
- Hans G.E. Hendriks, M.D., Ph.D. Department of Orthopedic Surgery & Trauma, Máxima MC, Eindhoven

Participating hospitals in the RCT and NE.

- Department of Orthopedic and Trauma Surgery, ETZ, Tilburg
- Department of Orthopedic and Trauma Surgery , Zuyderland Medical Center, Heerlen, Sittard-Geleen
- Department of Orthopedic and Trauma Surgery, OLVG, Amsterdam
- Department of Orthopedic Surgery, OCON, Hengelo
- Department of Orthopedic Surgery, IJsselland, Capelle aan de Ijssel

© 2024 Tol MCJM et al. *JAMA Network Open.*

Participating hospitals in the NE only:

- Department of Trauma Surgery, Spaarne Gasthuis, Haarlem
- Department of Orthopedic and Trauma Surgery, Antonius Ziekenhuis, Utrecht
- Department of Trauma Surgery, Noord-West Ziekenhuis, Alkmaar
- Department of Orthopedic Surgery, UMCG, Groningen
- Department of Trauma Surgery, Amsterdam UMC, Amsterdam
- Department of Orthopedic and Trauma Surgery, Isala, Zwolle
- Department of Trauma Surgery, Flevoziekenhuis, Almere
- Department of Orthopedic Surgery, Meander MC, Amersfoort
- Department of Orthopedic and Trauma Surgery, Máxima MC, Eindhoven

© 2024 Tol MCJM et al. *JAMA Network Open.*

eMethods. Data Fusion

Missing data

Missing data were prevalent in both the RCT and the NE data sets, concerning mainly the primary outcome and the covariate BMI. For example, in the NE data set, 35% of patients' primary outcome and 11% of patients' BMI were missing. See Fig. S1 for detailed missingness patterns. Missingness was negligible in the other covariates.

The outcomes were mainly thought to be missing by non-response to the post trial questionnaire, which was attributed to dementia, living status and mobility. Indeed, a logistic regression of the missingness indicator of the primary outcome on the covariates resulted in a statistically significant positive coefficient on mobility (p-value 0.002) in the RCT and a statistically significant positive coefficient on dementia (p-value 0.004) in the NE. Therefore, the outcomes were considered to be missing at random given the confounders, thus indicating that no bias will be introduced to the average treatment effect when missing outcomes are ignored in the analysis.

All missing data in the confounders were imputed with Classification And Regression Trees[1] using the R package MICE[2]. The outcome and treatment variables were not used as predictors for imputing the missing confounder values. The statistical analysis was then performed only on the subjects with an observed outcome.

Estimator

Augmented Inverse Probability Weighting (AIPW) was deployed to estimate the average treatment effect. AIPW requires model specifications for the (i) propensity score, i.e. the conditional probability of receiving PLA given the covariates age, gender, BMI, living status, baseline ASA scores, mobility and dementia; and (ii) conditional mean of the outcome variable given the same covariates. Flexible modeling strategies were chosen: the propensity score and outcome regression were modelled using an ensemble of methods, including elastic net regression[3], generalized linear models[4], generalized additive models[5], generalized boosted models[67] and multivariate adaptive regression splines[8]. These were implemented in the R package SuperLearner[9]. All this was performed using 10-fold cross-validation.

Data fusion

Besides unconfoundedness, which was assumed, another condition is necessary for data fusion. Namely, there should be no significant difference between the patients in the NE and the RCT in any of the treatment groups when correcting for the confounders. This condition was tested on one separately imputed combined dataset similarly to Lu et. Al. (2019)[10]. Specifically, a linear model was fitted for the EQ-5D-5L index at 6 months as a function of an experiment indicator and the confounding variables, separately in both groups of surgical approaches. The experiment indicator was equal to zero for patients in the RCT and one for patients in the NE. For both surgical approaches, the estimated coefficient belonging to the experiment indicator was close to zero with a p-value larger than 0.05, indicating no statistically significant effect of the experiment indicator on the outcome while keeping all other confounders constant. The test suggested that there was no evidence against data fusion, as there was no significant difference between the groups in the different experiments when all other confounders were included in the model. Accordingly, the data sets were combined, and the average treatment effect was estimated with AIPW as indicated in the protocol paper.[11]

Secondary outcomes

For the secondary outcomes, the estimates should only be viewed as exploratory because of multiple testing errors and because the outcomes had small variance (for instance, only 8 patients had a fall incident out of 281 in the NE). For AIPW, only an ensemble of generalized linear models, generalized additive models and multivariate adaptive regression splines were used to estimate the propensity score and outcome regression to limit runtime of the code. When the outcome was dichotomous, tests against data fusion were performed using logistic regression. All fusion tests indicated no evidence against data fusion, except Katz score, mobility, pain mean and pain max variables. For all these variables, the fused data sets were still analyzed, but only as a sensitivity analysis to check robustness of the study conclusions.

Sensitivity analysis

For predicting missing values by multiple imputation, CART was used as a predictive model for all confounders[12]. The findings were robust to the choice of predictive models (predictive mean matching and

© 2024 Tol MCJM et al. *JAMA Network Open.*

classification trees for binary, and linear regression and regression trees for continuous variables). The conclusions remained the same when the outcome was also imputed. These robustness findings held irrespective of whether BMI, the covariate with the highest missing percentage (11% in the NE data set), was included in the analysis. Using an ensemble of methods to model the components of the AIPW estimator rendered the results robust to modelling choices. Fitting individual, instead of ensemble, models led to no changes in the conclusions.

Software

Analysis was performed in R. Versions of R and the packages can be found below

Package	Version
R	4.1.3
foreign	0.8.83
Rcpp	1.0.9
tidyverse	1.3.2
encryptr	0.1.3
withr	2.5.0
mice	3.14.0
AIPW	0.6.3.2
SuperLearner	2.0.28
gbm	2.1.8.1
gam	1.20.2
earth	5.3.1
glmnet	4.1.4
foreach	1.5.2
doParallel	1.0.17
doSNOW	1.0.20

© 2024 Tol MCJM et al. *JAMA Network Open.*

eAppendix 2. Data Fusion Test for the Primary Outcome EQ-5D-5L Utility Score

```
[1] "===================================================================="
[1] "Direct Lateral Approach"
[1] "===================================================================="
                    term      estimate   std.error  statistic       df      p.value
1            (Intercept)  0.6604398920 0.218359719  3.0245500 290.7823 2.712350e-03
2       NERE_indicatorRE  0.0103088600 0.035277330  0.2922234 309.4809 7.703117e-01
3    BSL_Mobility_dicho1 -0.2296335543 0.037806397 -6.0739338 229.0922 5.146488e-09
4 BSL_LivingStatus_dicho1 -0.0955672207 0.050561892 -1.8901037 289.3977 5.974322e-02
5              ASA_dicho1 -0.1084115302 0.036087107 -3.0041624 276.6259 2.907060e-03
6         Dementia_dicho1 -0.2237989547 0.048229038 -4.6403363 305.4754 5.168386e-06
7             BSL_Gender1 -0.0043565757 0.033843039 -0.1287289 308.1923 8.976562e-01
8                 BSL_Age  0.0010336238 0.002236909  0.4620767 311.3296 6.443490e-01
9                 BSL_BMI  0.0009187454 0.004311558  0.2130889 218.1529 8.314566e-01
[1] "===================================================================="
[1] "Postero Lateral Approach"
[1] "===================================================================="
                    term     estimate   std.error  statistic       df      p.value
1            (Intercept)  1.255263275 0.274458984  4.5735915 251.4863 7.530240e-06
2       NERE_indicatorRE -0.062543620 0.042030732 -1.4880450 273.6287 1.378905e-01
3    BSL_Mobility_dicho1 -0.142878167 0.044295464 -3.2255711 234.0806 1.436706e-03
4 BSL_LivingStatus_dicho1 -0.119554908 0.059403907 -2.0125765 269.5273 4.515373e-02
5              ASA_dicho1 -0.063187017 0.043275243 -1.4601193 239.5928 1.455673e-01
6         Dementia_dicho1 -0.173657071 0.054822653 -3.1676152 269.7027 1.713662e-03
7             BSL_Gender1 -0.018725917 0.040444395 -0.4630040 270.7629 6.437334e-01
8                 BSL_Age -0.005677388 0.002641269 -2.1494922 271.7434 3.247835e-02
9                 BSL_BMI -0.001608018 0.005202790 -0.3090683 198.3985 7.575939e-01
```

© 2024 Tol MCJM et al. *JAMA Network Open.*

eFigure 1. Missing Values for Relevant Patient Characteristics and Outcomes

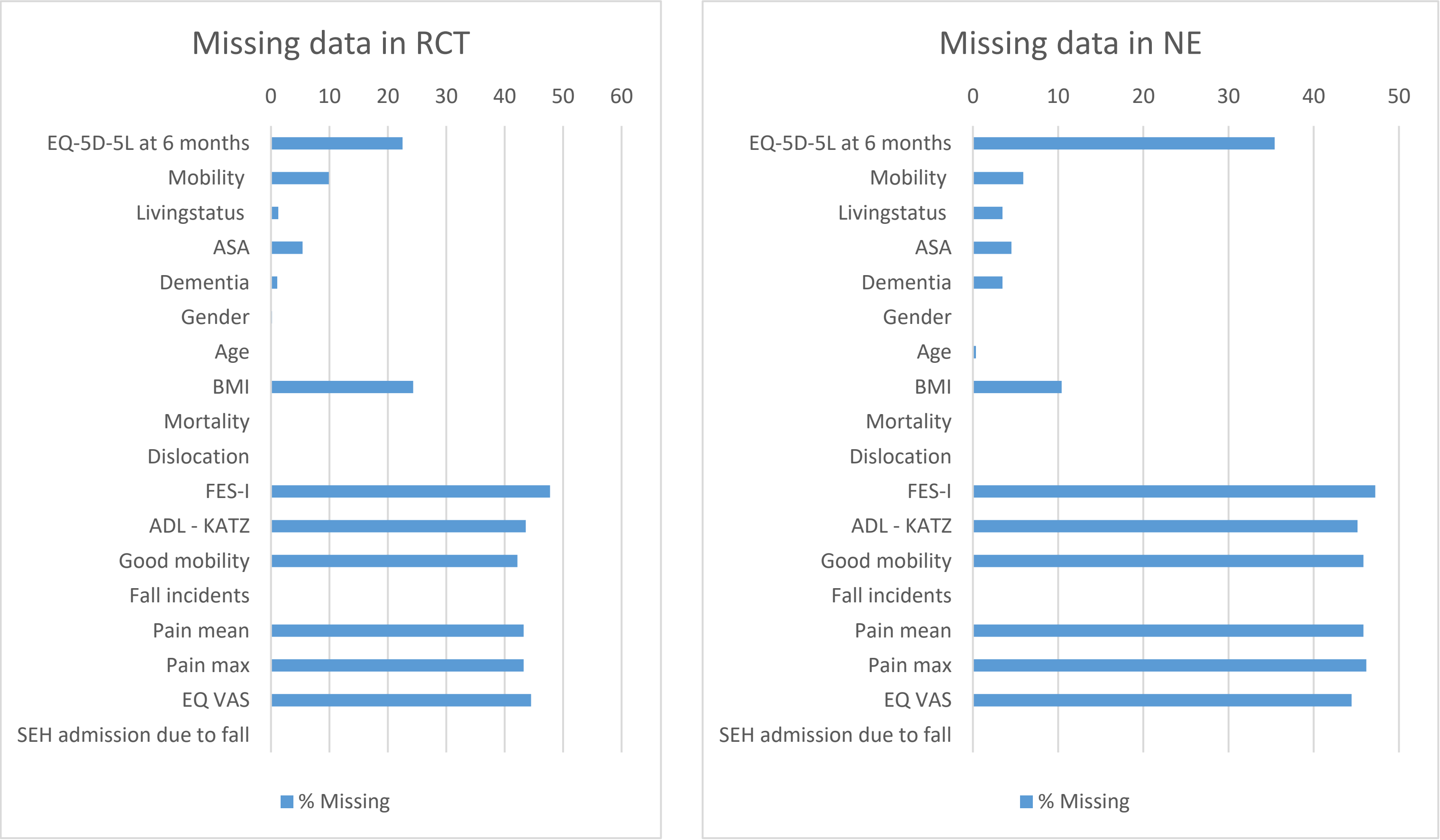

© 2024 Tol MCJM et al. *JAMA Network Open.*

eTable 1. Overview of Outcomes and Measurement Moments

Variable	Description	Baseline	4 weeks	3 months	4 months	6 months
Baseline characteristics	Including age, gender, BMI, comorbidities, living status, ASA, prescribed medication	RCT / NE				
Peri- postoperative outcomes	Including length of stay, surgery time, blood loss, discharge destination	RCT / NE				
EQ-5D-5L(1)	Health-related quality of life states consisting of five dimensions (mobility, self-care, usual activities, pain/discomfort and anxiety/depression) that are scored on 5 levels (no problems, slight problems, moderate problems, severe problems and extreme problems)	RCT / NE		RCT / NE		RCT / NE
Katz ADL(2)	Activities of Daily Living (ADL) functionality as introduced by Katz, resulting in a score ranging from 0 (ADL independent) to 6, (ADL dependent)	RCT / NE		RCT / NE		RCT / NE
Mobility score(3)	5 item mobility score(4), ranging from 0, indicating no walking aids to 5, indicating no functionality of lower extremity.	RCT / NE		RCT / NE		RCT / NE
Health-related and societal costs	Cost questionnaires to assess the use of healthcare resources and informal care as well as productivity-losses from unpaid and paid work (i.e. absenteeism and presenteeism). All resource use will be valued in accordance with the "Dutch Manual of Costing".(5)		RCT	RCT		RCT
Pain(6)	Numerical Rating Scale (NRS) ranging from 0 (no pain) to 10 (worst imaginable pain) for mean and maximum pain over the week			RCT / NE		RCT / NE
Fear of falling(7)	Falls Efficacy Scale International (FES-I), resulting in a score of 16, (no concern about falling) to 64 (severe concern about falling)			RCT / NE		RCT / NE
Tendency to fall	Number of falls, additional injuries as a result of falling, as reported by patients and/or retrieved from hospital charts			RCT / NE		RCT / NE
Complications	Re-interventions and (surgical) complications as reported by patients, and/or retrieved from hospital charts.			RCT / NE		RCT / NE
Mortality	As reported by patients' contact person and/or retrieved from hospital charts					RCT / NE
Short Physical Performance Battery test (SPPB)(8)	SPPB is a group of measures that combines the results of the gait speed, chair stand and balance tests. Each segment has a maximum of 4 points and the total score has a maximum of 12 points, high scores suggest better physical performance.(28)				RCT*	

*a subgroup of 70 patients will perform the SPPB test

© 2024 Tol MCJM et al. *JAMA Network Open.*

eTable 2. Baseline Characteristics of Lost to Follow Up Patients for Primary Outcome at Endpoint

	DLA<br>n = 58	PLA<br>n = 67
Sex, n (%)		
Female	36 (62)	46 (68)
Age, years	82 (8)	82 (7)
Dementia		
Evident + possibly, n/N (%)	23/56 (41)	17/66 (26)
ASA, n (%)	n = 57	n = 64
I	1 (2)	2 (3)
II	24 (42)	24 (38)
III	30 (53)	38 (59)
IV	2 (4)	0
BMI, mean (SD)	23.5 (4)	24.7 (4)
Katz, mean (SD)	1.9 (1.9)	1.1 (1.2)
Comorbidities, n (%)		
Cardial,		
MCI	5 (9)	10 (15)
DC	3 (5)	6 (9)
Arrhythmia	5 (9)	8 (12)
Neurological		
Hemiparalyse	1 (2)	3 (4)
CVA	7 (12)	10 (15)
Parkinson	5 (9)	4 (6)
Epilepsy	2 (3)	0
Pulmonal		
COPD	7 (12)	15 (22)
Astma	4 (7)	4 (6)
Pre-fracture mobility, n (%)	n = 54	n = 57
Without aids	15 (28)	17 (30)
1 crutch	6 (11)	7 (12)
Walker	28 (52)	28 (49)
Outside with help	5 (9)	4 (7)
No mobility	0	1 (2)
Living status, n (%)	n = 57	n = 66
Independent	24 (42)	31 (47)
Independent with help	20 (35)	21 (32)
Residential care	5 (9)	8 (12)
Nursing home	8 (14)	4 (6)
Rehabilitation unit	0	2 (3)

© 2024 Tol MCJM et al. *JAMA Network Open.*

eTable 3. Detailed Between-Group Effects at 3 and 6 Months for Primary and Secondary Outcomes

Adjusted effects are based on mixed models for the RCT groups and on AIPW models for the NE group and the fused results.

3 months	DLA		PLA		Crude mixed model		Adjusted model	
	n	Mean	n	Mean	Group effect	95%CI	Group effect	95%CI
EQ-5D-5L								
RCT	192	0.49	172	0.52	-0.08	-0.17 – 0.01	-0.07	-0.14 – 0.01
NE	101	0.54	75	0.58	0.05	-0.04 – 0.15		
EQ VAS								
RCT	162	62.9	146	65.6	-1.69	-7.52 – 4.13	-1.72	-7.24 – 3.80
NE	90	66.6	67	69.3	-3.10	-9.28 – 3.07		
FES-I								
RCT	146	38.3	128	33.7	5.08	0.90 – 9.27	4.34	1.15 – 2.83
NE	82	35.9	65	34.5	-0.26	-4.99 – 4.48		
Pain mean								
RCT	155	2.50	138	2.43	0.14	-0.61 – 0.88	0.23	-0.51 – 0.96
NE	90	3.42	67	2.87	0.25	-0.53 – 1.03		
Pain max								
RCT	155	3.06	139	3.20	0.03	-0.82 – 0.89	0.11	-0.79 – 0.55
NE	90	4.18	67	3.63	0.23	-0.76 – 1.22		
ADL – Katz								
RCT	155	1.72	142	1.67	0.23	-0.15 – 0.60	0.18	-0.16 – 0.53
NE	91	1.36	67	1.61	-0.70	-0.69 – -0.25		

6 months	DLA		PLA		Crude mixed model		Adjusted model		Fused data	
	n	Mean	n	Mean	Group effect	95%CI	Group effect	95%CI	Group effect	95%CI
EQ-5D-5L									0.00	-0.04 – 0.05
RCT	225	0.50	205	0.49	-0.05	-0.14 – 0.04	-0.04	-0.11 – 0.04		
NE	103	0.53	80	0.57	0.02	-0.08 – 0.11	0.06	-0.07 – 0.18		
EQ VAS									0.93	-2.63 – 4.50
RCT	179	67.9	165	65.6	0.30	-5.46 – 6.06	0.22	-5.24 – 5.67		
NE	89	66.6	69	67.6	1.62	-3.05 – 6.29	4.19	-6.85 – 15.23		
FES-I									-1.86	-4.45 – 0.72
RCT	152	36.8	138	34.0	5.59	-10.10 – 21.28	8.41	-6.65 – 23.48		
NE	86	34.5	66	34.7	-1.89	-6.64 – 2.85	-2.84	-15.33 – 9.65		
Pain mean										
RCT	163	2.14	153	2.53	-0.39	-1.13 – 0.35	-0.30	-1.03 – 0.44		
NE	89	3.42	67	3.04	0.49	-0.17 – 1.16	-0.89	-1.99 - 0.20		
Pain max										
RCT	165	2.69	151	2.98	0.03	-0.81 – 0.87	0.12	-0.72 – 0.96		
NE	88	4.36	67	3.76	0.72	-0.11 – 1.55	-1.16	-2.54 – 0.22		
ADL – Katz										
RCT	160	1.38	154	1.81	-0.02	-0.39 – 0.35	-0.08	-0.42 – 0.26		
NE	91	1.18	67	1.34	-0.44	-0.90 – 0.18	-0.21	-1.32 – 0.90		

© 2024 Tol MCJM et al. *JAMA Network Open.*

eFigure 2. Secondary Outcomes at 6-months

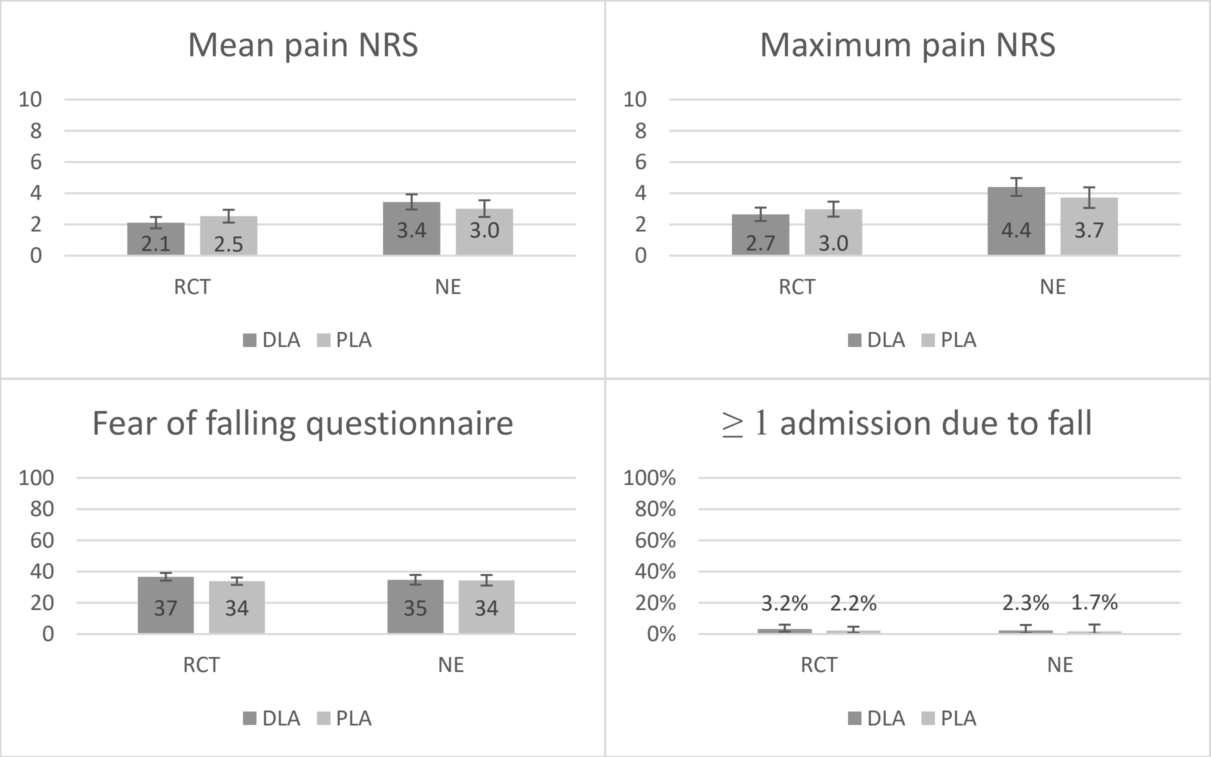

© 2024 Tol MCJM et al. *JAMA Network Open.*

eTable 4. Peri- and Post-Operative Outcomes of the RCT

	DLA (n = 283)	PLA (n = 272)	P value
Primary surgeon, n (%)			
Consultant	147 (52)	133 (49)	0.415
Resident	129 (46)	129 (47)	
Missing	7 (2)	10 (4)	
Time to surgery			
Days, mean (SD)	0.95 (1)	1.04 (1)	0.31
Surgery time, min, mean (SD)	57 (16)	59 (14)	0.29
Blood loss, cc, mean (SD)	259 (168)	253 (127)	0.72
Postoperative complication, n (%)			
Pulmonary embolism	4 (1)	2 (1)	0.69
Neuropraxie	2 (1)	1 (0)	1
Anemia with transfusion	18 (6)	20 (7)	0.74
Pneumonia	15 (5)	18 (7)	0.59
DAIR due to infection	3 (1)	4 (1)	0.72
DAIR due to hematoma	1 (0)	0	1
Superficial infection	1 (0)	0	1
Dislocation	0	2 (1)	0.24
Urinary tract infection	12 (4)	7 (3)	0.28
Bladder retention	9 (3)	2 (1)	0.06
Delirium	32 (11)	36 (13)	0.52
In hospital mortality, n (%)	13 (5)	9 (3)	0.52
Fall incident during admission, n (%)	7 (2)	6 (2)	0.85
Additional injury, n (%)	1 (0)	1 (0)	1
Length of hospital stay, days (SD)	8.3 (6)	8.1 (6)	0.76
Discharge destination, n (%)			
Independent	12 (4)	9 (3)	0.95
Independent with help	59 (21)	62 (23)	
Retirement home	21 (7)	18 (7)	
Nursing home	35 (12)	37 (14)	
Rehabilitation home	142 (50)	134 (49)	
Other	13 (5)	10 (4)	
Missing	1 (0)	2 (1)	
Mobility at discharge, n (%)			
Without aids	1 (0)	2 (1)	0.05
1 crutch	2 (1)	0	
Walker	73 (26)	97 (36)	
Outside with help	177 (63)	149 (55)	
No mobility	19 (7)	17 (6)	
Missing	11 (4)	4 (1)	

© 2024 Tol MCJM et al. *JAMA Network Open.*

eTable 5. Dislocation Rates

Dislocation	DLA	PLA	p-value
**Dislocation**			
RCT, number/n (%)	1/284 (0.4)	15/275 (5.5)	<0.01
NE, number/n (%)	2/175 (1.1)	6/113 (5.3)	0.06
Total, number/n (%)	3/459 (0.7)	21/388 (5.4)	<0.01
**Recurrent dislocation**			
RCT, number/n (%)	0	6/272 (2.2)	0.01
NE, number/n (%)	1/175 (0.6)	3/113 (2.7)	0.31
Total, number/n (%)	1/459 (0.2)	9/388 (2.3)	<0.01
**Revision due to dislocation**			
RCT, number/n (%)	0	8/272 (2.9)	<0.01
NE, number/n (%)	1/114 (0.9)	3/174 (1.7)	0.30
Total, number/n (%)	1/459 (0.2)	11/388 (2.8)	<0.01

© 2024 Tol MCJM et al. *JAMA Network Open.*

eTable 6. Reoperations by Indication

Indication for reoperation	RCT DLA (n=283)	RCT PLA (n=272)	NE DLA (n=172)	NE PLA (n=116)
Dislocation (revision to THA)	0	8	0	3
Infection	10	14	5	2
Any type of fracture	5	7	0	0
Other	3	6	2	4
Total number of reoperations (1-3 per patient)	18	35	7	9

Abbreviations: THA = total hip arthroplasty.

© 2024 Tol MCJM et al. *JAMA Network Open.*

eTable 7. Subgroup Analyses

	Dementia	
	**Yes**	**No**
**EQ-5D-5L**		
DLA	0.27 (0.18-0.37)	0.56 (0.50-0.61)
PLA	0.27 (0.19-0.35)	0.57 (0.51-0.62)
**Mixed model results**		
**Between groups**	**Effect (95% CI)**	**p-value**
**Surgical approach (DLA vs. PLA)**	-0.02 (-0.231, 0.181) *	0.81
**(Suspected) dementia (yes/no)**	0.29 (0.119, 0.465) [T]	<0.01
**Interaction approach X dementia**	-0.01 (-0.236, 0.211)	0.91

*in favor of PLA
[T] in favor of no dementia

	Experience	
	**Surgeon**	**Resident**
**EQ-5D-5L**		
DLA	0.48 (0.41-0.55)	0.54 (0.47-0.60)
PLA	0.47 (0.40-0.54)	0.51 (0.44-0.58)
**Mixed model results**		
**Between groups**	**Effect (95% CI)**	**p-value**
**Surgical approach (DLA vs. PLA)**	-0.08 (-0.205, 0.05)*	0.23
**Experience (yes/no)**	-0.02 (-0.09, 0.04)[T]	0.47
**Interaction approach X Experience**	0.04 (-0.045, 0.135)	0.33

* in favor of PLA
[T] in favor of resident

	Dislocation, n (%)		
	**PLA**	**DLA**	P value
Dementia			
Yes	5 out of 63 (8%)	0 out of 74 (0%)	0.02
No	10 out of 205 (4.8%)	1 out of 207 (0.5%)	<0.01
Experience			
Surgeon	6 out of 132 (4.5%)	0 out of 147 (0%)	0.01
Resident	8 out of 129 (6.2%)	0 out of 129 (0%)	<0.05

© 2024 Tol MCJM et al. *JAMA Network Open.*

eReferences

[1] Breiman, L. (1984). Classification and Regression Trees (1st ed.). Routledge. https://doi.org/10.1201/9781315139470

[2] Stef van Buuren and Karin Groothuis-Oudshoorn. mice: Multivariate imputation by chained equations in r. Journal of Statistical Software, 45(3), 2011. doi:10.18637/jss.v045.i03. URL https://doi.org/10.18637/jss.v045.i03.

[3] Hui Zou and Trevor Hastie. Regularization and variable selection via the elastic net. Journal of the Royal Statistical Society: Series B (Statistical Methodology), 67(2):301–320, 2005. doi: https://doi.org/10.1111/j.1467-9868.2005.
00503.x. URL https://rss.onlinelibrary.wiley.com/doi/abs/10.1111/j.1467-9868.2005.00503.x.

[4] J. A. Nelder and R. W. M. Wedderburn. Generalized linear models. Journal of the Royal Statistical Society. Series A (General), 135(3):370–384, 1972. ISSN00359238. URL http://www.jstor.org/stable/2344614.

[5] Trevor Hastie and Robert Tibshirani. Generalized Additive Models. Statistical Science, 1(3):297 – 310, 1986. doi: 10.1214/ss/1177013604. URL https://doi.org/10.1214/ss/1177013604.

[6] Jerome H. Friedman. Greedy function approximation: A gradient boosting machine. The Annals of Statistics, 29(5):1189 – 1232, 2001. doi: 10.1214/aos/1013203451. URL https://doi.org/10.1214/aos/1013203451.

[7] Jerome H. Friedman. Stochastic gradient boosting. Computational Statistics Data Analysis, 38(4):367–378, 2002. ISSN 0167-9473. doi: https://doi.org/10.1016/S0167-9473(01)00065-2. URL https://www.sciencedirect.com/
science/article/pii/S0167947301000652. Nonlinear Methods and Data Mining.

[8] Jerome H. Friedman. Multivariate Adaptive Regression Splines. The Annals of Statistics, 19(1):1 – 67, 1991. doi: 10.1214/aos/1176347963. URL https:
//doi.org/10.1214/aos/1176347963.

[9] Mark J. van der Laan, Eric C Polley, and Alan E. Hubbard. Super learner. Statistical Applications in Genetics and Molecular Biology, 6(1), 2007. doi:doi:10.2202/1544-6115.1309. URL https://doi.org/10.2202/1544-6115.1309.

[10] Yi Lu, Daniel O. Scharfstein, Maria M. Brooks, Kevin Quach, and Edward H. Kennedy. Causal inference for comprehensive cohort studies, 2019. URL https://arxiv.org/abs/1910.03531.

[11] Maria C J M Tol, Nienke W Willigenburg, Hanna C Willems et al. Postero-lateral or direct lateral approach for cemented hemiarthroplasty after femoral neck fracture (APOLLO): protocol for a multicenter randomized controlled
trial with economic evaluation and natural experiment alongside. Acta Orthop, 93:732–738, September 2022.

[12] Dekkers Olaf M. le Cessie Saskia Choi, Jungyeon. A comparison of different
methods to handle missing data in the context of propensity score analysis. European Journal of Epidemiology, 34(1):23 – 36, 2019. doi: 10.1007/
s10654-018-0447-z. URL https://doi.org/10.1007/s10654-018-0447-z.
