# Supplementary material for: Posterolateral or Direct Lateral Surgical Approach for Hemiarthroplasty After a Hip Fracture: A Randomized Clinical Trial Alongside a Natural Experiment
Source: JAMA Netw Open. 2024 Jan 11;7(1):e2350765. doi: 10.1001/jamanetworkopen.2023.50765 (PMC10784859; doi:10.1001/jamanetworkopen.2023.50765)
Supplement: Supplement 3. — APOLLO Research Group Members [file jamanetwopen-e2350765-s003.pdf]

Supplemental Online Content: Nonauthor Collaborators
*First name, last name, and suffix (if applicable) are required and will appear in PubMed.

*Group Name(s): APOLLO research group							
***First Name and Middle Initial(s)**	***Last Name**	***Suffix (eg, Jr, III)**	Academic Degrees	Institution	Location (city, state/province, country)	Role or Contribution, eg, chair, principal investigator	Group (if more than 1 Group listed in the byline) and/or Subgroup (eg, Steering Committee)
Frank	van Roon		MD	ETZ	Tilburg	Data aquisition	APOLLO research group
Martijn	van Dijk		MD, PhD	Antonius Ziekenhuis	Utrecht	Data aquisition	APOLLO research group
Jort	Keizer		MD	Antonius Ziekenhuis	Utrecht	Data aquisition	APOLLO research group
Anne J.H.	Vochteloo		MD, PhD	OCON	Hengelo	Data aquisition	APOLLO research group
Pieter	Joosse		MD, PhD	Noord-West Ziekenhuis	Alkmaar	Data aquisition	APOLLO research group
Bert	Boonen		MD, PhD	Zuyderland Medical Center	Heerlen	Data aquisition	APOLLO research group
Jetse	Jelsma		MD, PhD	Zuyderland Medical Center	Heerlen	Data aquisition	APOLLO research group
Dieuwertje	Theeuwen		MD	Zuyderland Medical Center	Heerlen	Data aquisition	APOLLO research group
Joris J.W.	Ploegmakers		MD, PhD	UMCG	Groningen	Data aquisition	APOLLO research group
Tim	Schepers		MD, PhD	Amsterdam UMC	Amsterdam	Data aquisition	APOLLO research group
Evelien	van der Meij		MD, PhD	IJsselland	Capelle aan den IJssel	Data aquisition	APOLLO research group
Svenhjalmar H.	van Helden		MD, PhD	Isala	Zwolle	Data aquisition	APOLLO research group
Rutger	Zuurmond		MD, PhD	Isala	Zwolle	Data aquisition	APOLLO research group
Bart A.	van Dijkman		MD	Flevoziekenhuis	Almere	Data aquisition	APOLLO research group
Thomas D.	Berendes		MD, PhD	Meander MC	Amersfoort	Data aquisition	APOLLO research group
Hans G.E.	Hendriks		MD, PhD	Máxima MC	Eindhoven	Data aquisition	APOLLO research group
