# Supplementary material for: Posterolateral or Direct Lateral Surgical Approach for Hemiarthroplasty After a Hip Fracture: A Randomized Clinical Trial Alongside a Natural Experiment
Source: JAMA Netw Open. 2024 Jan 11;7(1):e2350765. doi: 10.1001/jamanetworkopen.2023.50765 (PMC10784859; doi:10.1001/jamanetworkopen.2023.50765)
Supplement: Supplement 4. — Data Sharing Statement [file jamanetwopen-e2350765-s004.pdf]

Data Sharing Statement

Tol. Posterolateral or Direct Lateral Surgical Approach for Hemiarthroplasty After a Hip Fracture. *JAMA Netw Open*. Published January 11, 2024.
doi:10.1001/jamanetworkopen.2023.50765

Data
Data available: Yes
Data types: Other (please specify)
Additional Information: Data available upon reasonably request
How to access data: JointResearch@olvg.nl
When available: With publication

Supporting Documents
Document types: Other (please specify)
Additional Information: Data available upon reasonably request
How to access documents: JointResearch@olvg.nl
When available: With publication

Additional Information
Who can access the data: Researches requesting the data
Types of analyses: for any purpose
Mechanisms of data availability: after approval of a proposal
